# A Small RNA Derived From the 5′ End of the IS200 *tnpA* Transcript Regulates Multiple Virulence Regulons in *Salmonella typhimurium*

**DOI:** 10.1111/mmi.70016

**Published:** 2025-08-13

**Authors:** Ryan S. Trussler, Naomi‐Jean Q. Scherba, Hoda Kooshapour, Michael J. Ellis, Konrad U. Förstner, Matthew Albert, Alexander J. Westermann, David B. Haniford

**Affiliations:** ^1^ Department of Biochemistry University of Western Ontario London Ontario Canada; ^2^ Department of Microbiology, Biocenter University of Würzburg Würzburg Germany; ^3^ Helmholtz Institute for RNA‐Based Infection Research (HIRI), Helmholtz Centre for Infection Research (HZI) Würzburg Germany; ^4^ BenchSci Toronto Ontario Canada; ^5^ ZB MED, Information Centre for Life Sciences Cologne Germany

**Keywords:** post‐transcriptional gene regulation, salmonella pathogenesis, small RNA, SPI‐1 regulation, transposon domestication

## Abstract

The insertion sequence IS200 is widely distributed in Eubacteria. Despite its prevalence, IS200 does not appear to be mobile and as such is considered an ancestral component of bacterial genomes. Previous work in *Salmonella enterica* revealed that the IS200 *tnpA* transcript is processed to form a small, highly structured RNA (*5*′*tnpA*) that participates in the posttranscriptional control of *invF* expression, encoding a key transcription factor in this enteropathogen's invasion regulon. To further examine the scope of *5′tnpA* transcript integration into *Salmonella* gene expression networks, we performed comparative RNA‐seq, revealing the differential expression of over 200 genes in a *Salmonella* SL1344 *5′tnpA* disruption strain. This includes the genes for the master regulators of both invasion and flagellar regulons (HilD and FlhDC, respectively), plus genes involved in cysteine biosynthesis and an operon (*phsABC*) encoding a thiosulfate reductase complex. These expression changes were accompanied by an 80‐fold increase in *Salmonella* invasion of HeLa cells. Follow‐up experimentation suggested an additional direct target of *5′tnpA* to be the small RNA PinT, which has previously been shown to be a negative regulator of invasion genes through its inhibitory action on key transcription factors governing the *Salmonella* pathogenicity island 1 regulon. This study provides a powerful new example of bacterial transposon domestication that is based not on the production/use of a regulatory protein or regulatory DNA sequences, but on the function of a transposon‐derived small RNA.

## Graphical abstract

We show deletion of an IS200‐encoded sRNA (5′tnpA) in Salmonella impacts the expression of SPI‐1, flagellar, cysteine, and phsABC regulons. Evidence is presented that, with regard to the SPI‐1 regulon, 5′tnpA may function through base pairing with another sRNA known to regulate SPI1 called PinT.

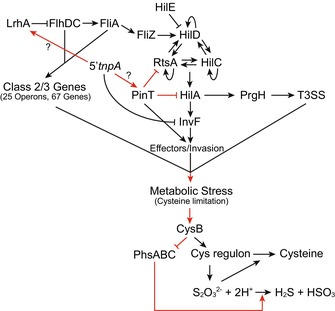

## 1 Introduction

The Gram‐negative bacterium *Salmonella enterica* serovar Typhimurium (hereafter referred to as *Salmonella*) is a major cause of gastrointestinal diseases in humans and a wide range of agriculturally important animals (Scallan et al. 2011; Chaudhuri et al. 2013). *Salmonella* typically enters the GI tract through ingestion of contaminated food or water and initially infects epithelial cells of the lower intestine (Zhou and Galán 2001). Infection is dependent on flagellar‐mediated motility of *Salmonella* and the interaction between bacterial fimbria and host cell membranes, which promotes adhesion (Jones et al. 1992; Yue et al. 2012). The subsequent invasion of intestinal epithelial cells is directed by the induction of invasion genes, which are mostly encoded on *Salmonella* pathogenicity island 1 (SPI‐1), a horizontally acquired genetic element that comprises approximately 40 genes arranged in several operons (Mills et al. 1995). The SPI‐1 genes include the components of a Type III Secretion System (T3SS), effector proteins, and transcription factors (TF), which control expression of themselves as well as the T3SS and effector genes (Akbar et al. 2003). The T3SS transports *Salmonella* effector proteins into the host cell, altering its architecture in a manner that permits *Salmonella* to be taken up (Que et al. 2013). *Salmonella* can replicate inside host epithelial cells and, through these cells, gain access to the lymph system, where the bacterium can enter macrophages and replicate (Hurley et al. 2014).

A relatively large number of genes participate in host cell invasion, requiring precise spatiotemporal regulation of their expression along the infection cycle. Having the invasion genes turned on only when *Salmonella* is in an optimal position within the GI tract is expected to avoid a metabolic burden that otherwise could reduce the capacity of *Salmonella* to compete for nutrients with other microbes (Sturm et al. 2011). Indeed, spatiotemporal regulation of this process is achieved by the invasion genes being organized into a complex positive feed‐forward cascade involving several TFs. At a higher level of regulation, this cascade is responsive to several different two‐component signal transduction systems and other global regulators (Ellermeier et al. 2005; Erhardt and Dersch 2015). The TF HilD is positioned at the top of this cascade, receiving inputs from these signal transduction systems. HilD activates the transcription of two other TFs, HilC and RtsA, as well as of itself. HilC and RtsA also positively self‐regulate and control each other, establishing a strong positive feed‐forward loop. Upon surpassing a specific threshold of active protein, HilD then activates transcription of *hilA*, encoding a TF that activates transcription of additional TFs InvF and PrgH. The former controls expression of the effector gene operons, while the latter controls the expression of structural components of the T3SS (Golubeva et al. 2012). Besides, there is cross‐talk between *Salmonella* invasion and flagellar regulons. For example, the FliZ protein, which is a component of the flagellar regulon, binds directly to the *hilD* promoter and activates its transcription (Cott Chubiz et al. 2010). In another linkage, HilD acts as a positive regulator of *flhDC* expression, with FlhDC being the master regulator of the flagellar regulon. Expression of the *flhDC* operon is negatively regulated by a number of TFs including LrhA, YdiV, SlyA, RcsB, RtsB, and RflM, and also positively controlled by Crp‐cAMP (Mouslim and Hughes 2014).

In addition to receiving regulatory input from proteins, *hilD* expression is subject to posttranscriptional control by small noncoding RNAs (sRNA). Bacterial sRNAs constitute a versatile class of regulators that can affect the expression of target genes through a variety of different mechanisms; for example, through modulating the activity of regulatory proteins or binding to complementary sequences in mRNAs, influencing their transcription, translation, or stability (Storz et al. 2011). For example, sRNAs CsrB and CsrC compete with other RNAs for binding the global translational repressor CsrA, effectively titrating CsrA protein and relieving *hilD* repression (Altier et al. 2000; Martínez et al. 2011; Vakulskas et al. 2015). Transcription of *csrB* and *csrC* is turned on by the TF SirA and negatively regulated by Crp‐cAMP (Teplitski et al. 2006; El Mouali et al. 2020). While CsrA binds to the *hilD* 5′UTR, this mRNA harbors an exceptionally long 3′UTR, which serves as a hub for Hfq‐dependent sRNAs (López‐Garrido et al. 2014; El Mouali et al. 2018; Abdulla et al. 2023). For example, Spot 42 (encoded by the gene *spf*) is another sRNA that impinges on *hilD* expression. Specifically, Spot 42, whose transcription is negatively regulated by Crp‐cAMP, acts as a positive regulator of *hilD* expression through a mechanism involving an unstructured region within the 3′UTR of the *hilD* transcript (El Mouali et al. 2018). Similarly, the RpoS‐dependent sRNA SdsR binds a distinct site within the *hilD* 3′UTR, also increasing *hilD* mRNA levels (Abdulla et al. 2023). In contrast, the sRNA PinT is a negative regulator of SPI‐1 and acts downstream of HilD in the SPI‐1 cascade (reviewed in Barquist et al. 2016). Specifically, transcriptionally activated through PhoPQ upon host cell invasion (Westermann et al. 2016), PinT binds and translationally represses the mRNAs encoding RtsA, HilA, and the SPI‐1 effectors SopE and SopE2 (Westermann et al. 2016; Kim et al. 2019), and sponges the host adherence and invasion‐associated sRNA InvS (Kooshapour et al. 2025).

**FIGURE 1:**
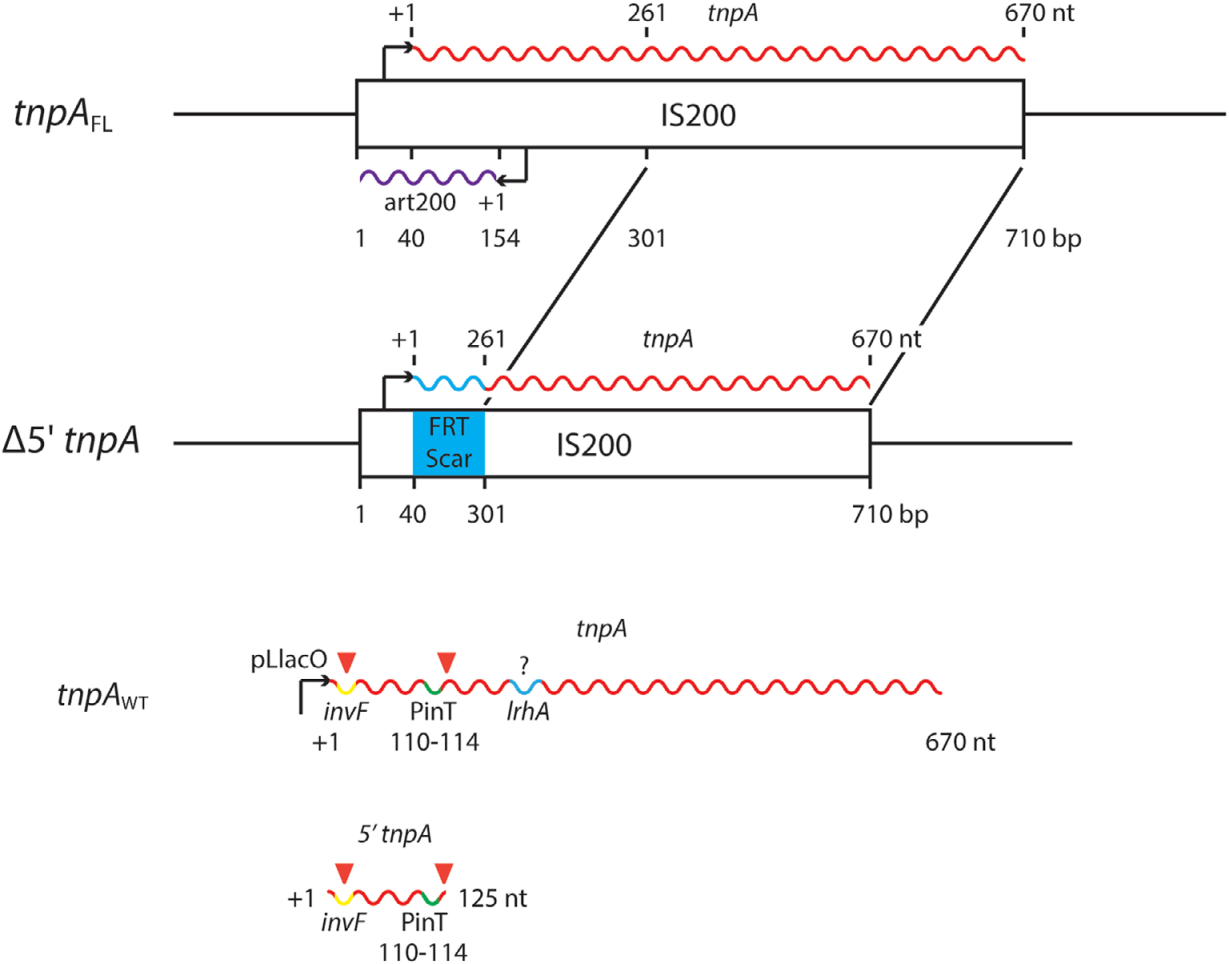
Structure of IS200 and the derived *5′tnpA* sRNA. The structure of IS200 is shown along with IS200‐encoded transcripts including the transposase transcript (red wavy line—*tnpA*) and the antisense transposase transcript (purple wavy line—*art200*). The IS200 insertion sequence is 710 bp in length and the transcription start site for *tnpA* (+1) is located at bp 40. A poorly defined promoter (bent arrow) for *tnpA* is located between bp 1 and 40. The designation Δ*5′tnpA* defines a 5′ internal deletion in IS200 in which the transcription start site of *tnpA* is fused to bp 301, removing 261 nt from the *tnpA* transcript. *FRT‐scar* refers to the Flip‐recombinase ‘scar’ sequence left after excision of antibiotic resistance genes used for selection in the construction of the Δ*5′tnpA* strain. Bottom diagram shows the full‐length *tnpA* gene/transcript (*tnpA*_WT_) fused to the IPTG‐inducible pLlacO‐1 promoter (present in pDH968). Yellow, green and blue segments are interaction sites for *invF* (identified in Ellis et al. 2017), PinT (identified in this study, see below) and a putative interaction site (indicated by?) for *lrhA* (predicted using the IntaRNA pairing tool (Mann et al. 2017)), respectively. The segment designated 110–114 within the PinT binding region contains 5 nucleotide exchanges introduced to abolish PinT binding (see text for details). Red triangles define *tnpA* endoribonucleolytic processing sites previously identified by TIER‐seq (Chao et al. 2017) or primer extension analysis (Ellis et al. 2017).

The acquisition of SPI‐1 and other pathogenicity islands via horizontal gene transfer has shaped the evolutionary trajectory of *Salmonella* species. Exogenous genetic elements contributing to horizontal gene transfer, including plasmids, prophages, and transposons, continue to impact *Salmonella* evolution by promoting genetic rearrangements that impact adaptation and fitness (Brockhurst et al. 2019). The small prokaryotic insertion sequence IS200 provides an interesting new example of this. It is widely conserved in Enterobacteriaceae and found throughout Eubacteria and Archaea. In a few bacteria where IS200 has been studied in detail, including *Escherichia coli* and *Salmonella*, this transposon has been found to be essentially immobile yet conserved in sequence and an active template for transcription, producing both a transposase (*tnpA*) mRNA and an antisense transcript (*art200*) that is divergently encoded to *tnpA* (Figure 1) (Beuzón and Casadesús 1997; Beuzón et al. 2004; Filée et al. 2007; Sittka et al. 2009; Kröger et al. 2012). Given the evidence that multiple independent mechanisms conspire to prevent translation of the *tnpA* transcript (Ellis et al. 2015), we previously proposed that one or more IS200‐derived transcript(s) might serve as sRNA(s) to regulate *Salmonella* gene expression. This led to the discovery that the *tnpA* transcript forms a highly structured and stable sRNA comprised of the first ~111–125 nucleotides of the transcript, which we refer to here as *5′tnpA*; *tnpA* endoribonucleolytic processing sites are shown in Figure 1. Moreover, we demonstrated previously that the mRNA of the SPI‐1 TF InvF, which controls effector gene expression, is a direct target of *5′tnpA*: overexpression of *5′*tnpA reduced *invF* expression, and disrupting all seven copies of IS200 in the pathogenic SL1344 strain of *Salmonella* (referred to here as the Δ*5′tnpA* strain) had the opposite effect (Ellis et al. 2017). Once induced, *invF* transcript levels greatly exceed those of *tnpA*, and accordingly, it is unlikely that *tnpA* regulation of *invF* would have a significant impact on SPI‐1 function after the system had been turned on. In contrast, early in growth before SPI‐1 induction, *tnpA* transcript levels significantly exceed *invF* mRNA levels, and it is thus likely that the *5′tnpA* sRNA interferes with SPI‐1 induction early in growth (Ellis et al. 2017). Interestingly, in a mouse infection model, the Δ*5′tnpA* strain exhibited reduced infectivity in GI tract tissues. This was somewhat counterintuitive because of the increased expression of *invF* in this strain, a positive regulator of invasion. However, as the Δ*5′tnpA* strain displays a slight growth defect in culture, we speculated that the reduced infectivity of this strain in tissues of the mouse GI tract may be due to a reduced ability to compete with other gut microbes for nutrients (Ellis et al. 2018).

In the current work, we have more extensively characterized the role of the IS200‐derived *5′tnpA* sRNA in *Salmonella* by performing comparative transcriptomics between Δ*5′tnpA* and wild‐type (WT) strains at multiple growth phases. We show that the deletion of *5′tnpA* from all seven copies of IS200 affects the expression of over 200 genes by 2‐fold or more. At least four major pathways are impacted by this absence, including the invasion and flagellar regulons, the cysteine regulon, and expression of a thiosulfate reductase. Regarding the SPI‐1 regulon, we provide evidence that *5′tnpA* negatively regulates the expression of the HilD‐HilC‐RtsA regulatory loop, possibly by affecting the function of the sRNA PinT. Further, we show that premature SPI‐1 and flagellar induction in *5′tnpA* deletion mutants of *Salmonella* impacts sulfur metabolism. Importantly, these *5′tnpA*‐dependent expression changes culminate in an invasion phenotype in an epithelial cell culture infection model. Together, this study implies a surprisingly widespread regulon for this transposon‐derived sRNA and corroborates *5′tnpA*'s role as an important posttranscriptional regulator of *Salmonella* invasion of host cells.

## 2 Results

### 2.1 Comparative RNA‐Seq Analysis Reveals Widespread Impact of *5′* tnpA on Salmonella Virulence Gene Expression

To better understand the function of the IS200 transposase transcript in the regulation of *Salmonella* genes, we removed the 5′ portion (nucleotides 41–300) of the *tnpA* gene from all seven copies of IS200 in the *Salmonella* SL1344 chromosome (Figure 1 and Figure S1). This was done by successive transduction of independently constructed disruption alleles into a single recipient strain (Ellis et al. 2017). The genome of the resulting strain was sequenced, corroborating the successful disruption of all seven IS200 copies and identifying only a single synonymous mutation (in the *ruvB* gene, encoding a Holliday junction DNA helicase). We refer to this strain as Δ*5′tnpA*. A complementation analysis wherein the WT *ruvB* gene was introduced in the Δ*5′tnpA* strain revealed that the *ruvB* mutation does not contribute to the phenotype of the Δ*5′tnpA* strain described below (Figure S2). We note, however, that the comparative gene expression analysis presented below was not done on the *ruvB*‐complemented strain.

**FIGURE 2:**
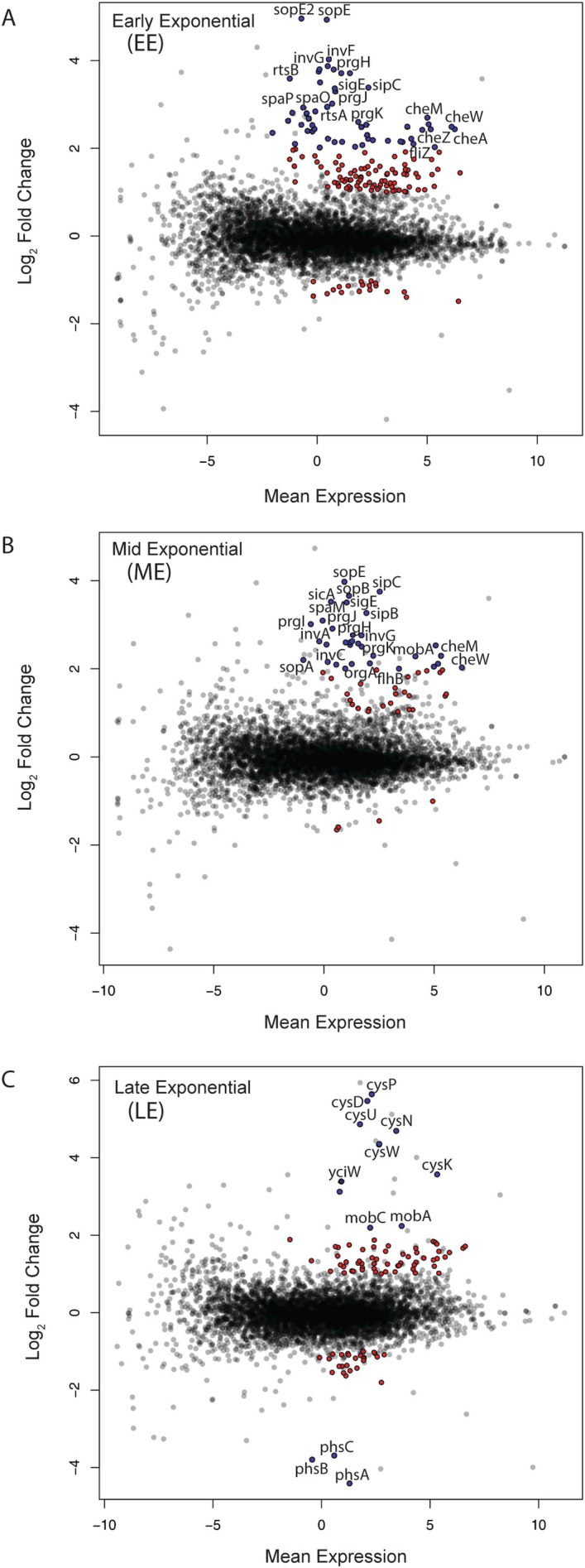
Differential gene expression analysis via RNA‐seq. (A‐C) MA plots summarizing comparative RNA‐seq data (Δ*5′tnpA* vs. wild‐type [WT]) for early (EE), mid (ME), and late (LE) exponential growth phases. Positive and negative fold‐changes mean up‐ or down‐regulations, respectively, in the mutant compared to the WT strain. For each growth phase, RNA was extracted from three clones of WT and Δ*5′tnpA* strains. Genes showing differential expression of 2‐fold or more were colored; in panels A and B, blue dots refer components of either SPI‐1 or flagellar regulons, whereas in panel C, blue dots represent components of either the cysteine regulon or the *phsABC* operon. Due to space limitations not all up‐ or down‐regulated genes in the affected regulons are labeled.

We performed comparative gene expression analysis by RNA‐seq using total RNA samples extracted from WT and Δ*5′tnpA* strains (each three biological replicates) grown in rich media (LB) at three growth stages; the transition from lag phase to early‐exponential phase (EE), mid‐exponential phase (ME), and the transition from late‐exponential to stationary phase (LE) (see Figure S3 for growth curves and Tables S1–S3 for RNA‐seq data). Across all growth stages, a total of 219 genes exhibited differential expression between WT and Δ*5′tnpA Salmonella* (FC ≥ |2|; *p* < 0.05) (Table S3; Figure 2A–C), most of which were upregulated in the Δ*5′tnpA* strain (88%, 94%, and 71% of differentially expressed genes for EE, ME, and LE, respectively), implying *5′tnpA* to mainly act as an inhibitor of target gene expression. In EE and ME phases, genes exhibiting the largest increases in expression in the Δ*5′tnpA* strain are involved in invasion (SPI‐1 genes), flagellar‐dependent cell motility, and chemotaxis (blue dots in Figure 2A,B; also see Figure S4A [heat map] and B [GO analysis]). In LE growth, genes involved in cysteine biosynthesis (cysteine regulon) were the most up‐regulated genes in the Δ*5′tnpA* strain (blue dots in Figure 2C and also see Figure S4A). Genes involved in thiosulfate reduction (components of the *phsABC* operon) were the most highly downregulated genes in the entire data set. EE phase had the highest, and ME the fewest, number of differentially expressed genes that are unique to a specific phase (84 or 6 genes, respectively) (Figure S4C [Venn diagram]). There were 21 differentially expressed genes present in all three growth phases, and the majority of these (67%) are constituents of the flagellar regulon. Overlapping differentially expressed genes in EE and ME (35 genes) are mainly genes in the invasion cascade (63%). Gene lists for each of these categories are presented in Table S4, and the full set of differentially expressed genes is shown in Figure S5.

Together, the RNA‐seq results support earlier published data implicating *5′tnpA* sRNA as a negative regulator of SPI‐1 (see Introduction). From the current work, further insight into how *invF* expression is affected by *5′tnpA*–that is, in addition to being directly targeted by the sRNA (Ellis et al. 2017)—can be inferred. That is, while our comparative transcriptomics approach cannot distinguish between direct, base‐pairing‐mediated regulations and indirect effects from deleting the *5′tnpA* sRNA, the RNA‐seq analysis identified four genes upstream of *invF* in the SPI‐1 regulatory cascade as being upregulated in the Δ*5′tnpA* compared to the WT strain early in growth, including *hilD* (2.3‐fold), *hilC* (2.9‐fold), *rtsA* (7.7‐fold), and *hilA* (7.7‐fold) (Figure S4A). Accordingly, the increased expression of the three genes (*hilD*, *hilC*, and *rtsA*) that make up the positive feed‐forward loop that activates the major positive regulator of *invF* transcription (HilA) could reflect a second mechanism by which *5′tnpA* is linked to *invF* expression. In line with the idea of two additive regulations (direct and indirect) converging at the level of InvF, the *invF* mRNA itself was strongly upregulated (16.3‐fold) in EE growth in the Δ*5′tnpA* strain (Figure S4A).

With respect to bacterial motility, genes for regulators (e.g., *flhDC* [3.1‐fold] and *fliA* [3.7‐fold]) and structural components of flagella (e.g., *flhB* [3.8‐fold], *flhA* [3.4‐fold], and *motB* [5.4‐fold]), genes involved in chemotaxis (e.g., *cheR* [4.7‐fold], *cheA* [5.4‐fold], and *aer* [2.9‐fold]), and the gene for a posttranslational activator of HilD (*fliZ*) [4.3‐fold]) were upregulated in the Δ*5′tnpA* strain in EE and ME phases of growth (Figure 2A,B and Figure S4A). Notably, both flagellar‐dependent motility and chemotaxis are important for invasion (Schmitt et al. 2001; Wozniak et al. 2009). In LE growth, differential expression of invasion genes was almost completely lost except for relatively small differences in *invR*, *sigE*, and *sopE2* (up to 3.3‐fold). This likely reflects the timely induction of invasion genes in the WT strain that is characteristic of this growth phase in rich media (Bustamante et al. 2008). In contrast to invasion genes, the increased expression of flagellar regulon genes observed in the earlier phases extends into LE growth (Figure S4A,B). Additionally, genes involved in sulfur assimilation/cysteine synthesis showed increased expression of the largest magnitude in the LE phase (Figure 2C and Figure S4A,B). This group comprises 13% of all differentially expressed genes in this growth phase. Components of the cysteine regulon (e.g., *cysP*, *cysD*, *cysU*, *cysN*, *cysW*, *cysC cysK*, and *yciW*) were amongst the most strongly upregulated genes in the entire data set. In contrast, genes involved in thiosulfate reduction (components of the *phsABC* operon) were amongst the most strongly downregulated genes in the data set (Figure S4A).

### 2.2 Independent Validation of 5′tnpA‐Dependent Expression Changes

**FIGURE 3:**
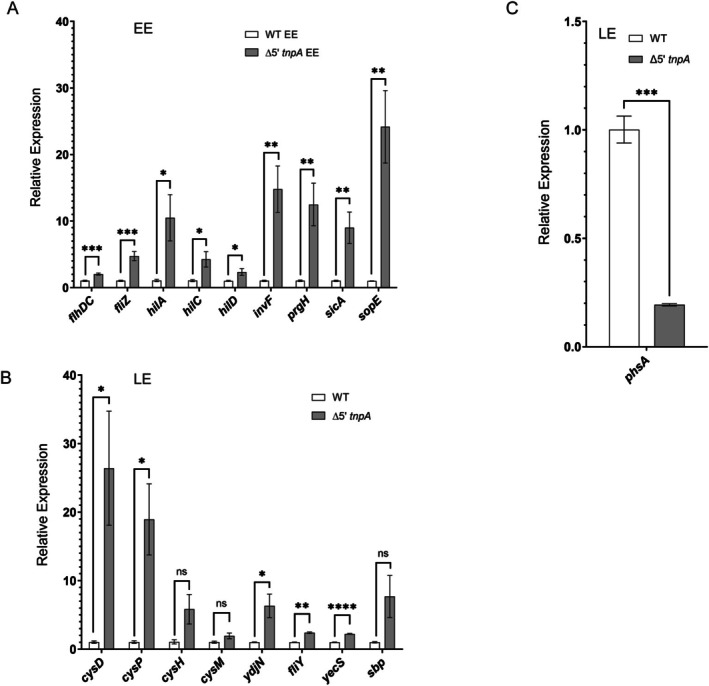
Validation by qRT‐PCR of select genes differentially expressed according to the RNA‐seq analysis. Total RNA was isolated from the indicated strains and subjected to qRT‐PCR analysis. In (A), strains were grown to EE phase and the targets for qRT‐PCR included select flagellar and SPI‐1 regulon genes. In (B) and (C), strains were grown to LE phase and targets included cysteine regulon and *phsABC* operon genes, respectively. For each pairwise comparison, the expression value for the WT strain was set to one. Bars refer to the mean and error bars show the standard deviation. *p* values (unpaired *T*‐test) are indicated as follows: Ns, not statistically significant (*p* ≥ 0.05); *, **, *** and ****p ≤ 0.05, 0.01, 0.001 and 0.0001, respectively. For A, B and C, 6, 3 and 3 independent clones were analyzed, respectively.

We used qRT‐PCR to validate the trends in differential gene expression for WT and Δ*5′tnpA* strains discussed above (Figure 3A–C). With the exception of the magnitude of the decrease in *phsA* expression (4‐fold in qRT‐PCR versus 21‐fold in the RNA‐seq analysis), the results show strong correlations between the two methods. The observed increase in *fliZ* expression in the Δ*5′tnpA* strain in both RNA‐seq and qRT‐PCR analyses could in principle be due to the genetic manipulation we performed at the *tnpA4* locus in the process of generating the Δ*5′tnpA* strain. That is, replacing the 5′ portion of this *tnpA* gene copy with the FLP‐scar sequence could theoretically impact the expression of neighboring genes, including the *fliA* gene which is adjacent to *tnpA4* (see Figure S1). The *fliA* gene encodes the FliA protein, also referred to as *σ* ^28^, and drives the expression of *fliZ* (Ikebe et al. 1999). Accordingly, if the disruption of *tnpA4* were to increase expression of *fliA*, this would increase FliZ levels, which in turn would increase HilD activity. We tested the impact of this disruption alone in an otherwise WT background on *fliZ* and *hilD* expression via qRT‐PCR and found no increase in expression of either of these genes (Figure S6). Thus, the disruption at the *tnpA4* locus was seemingly insufficient on its own for the gene expression profile of the Δ*5′tnpA* strain documented above.

### 2.3 5′tnpA Delays SPI‐1 Onset With Effects on Salmonella Invasion Into Epithelial Cells

**FIGURE 4:**
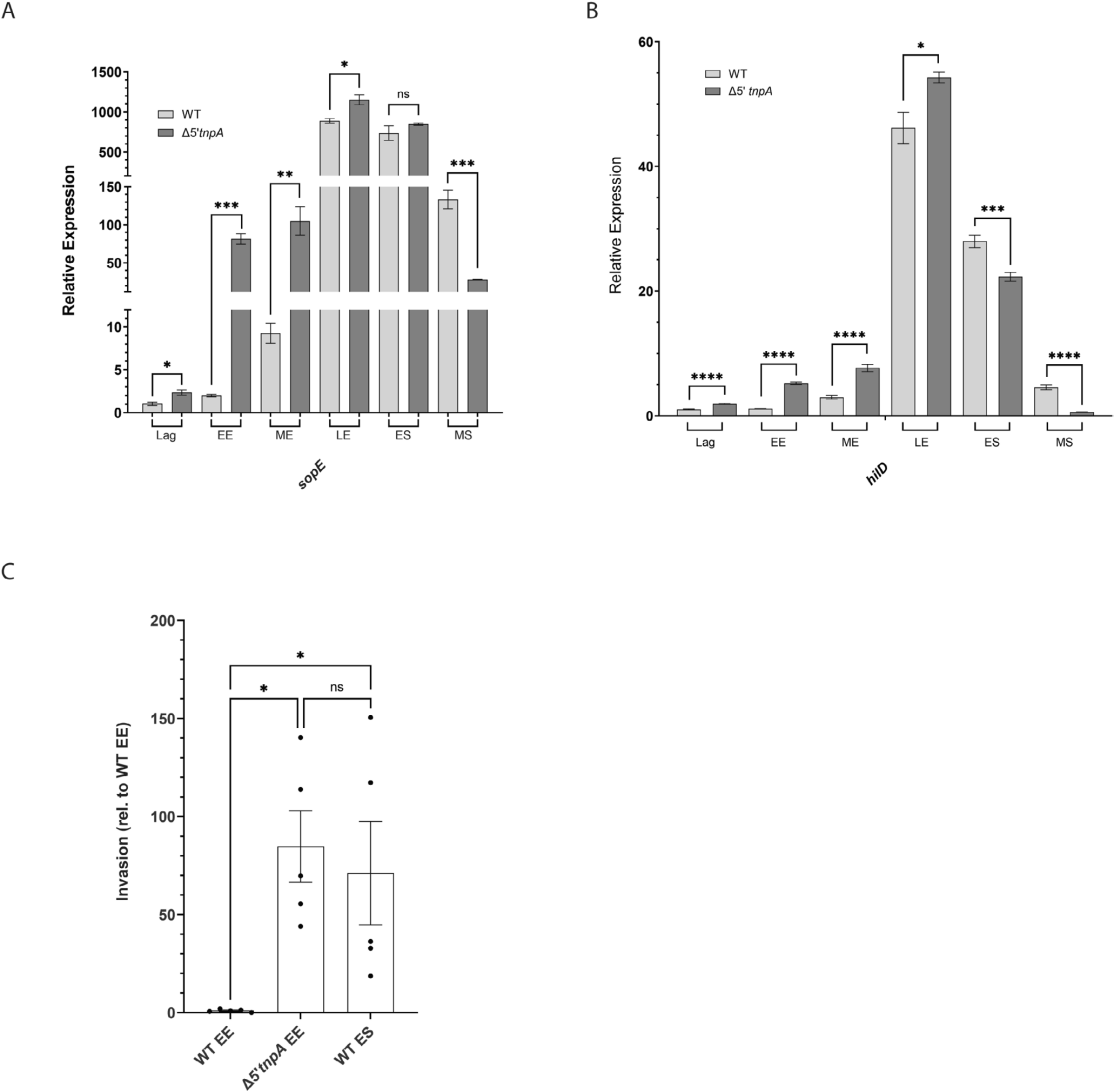
Impact of *5′tnpA* deletion on *sopE* and *hilD* expression over a time course of growth in rich medium and on invasion of HeLa cells. For each of the indicated growth points (Lag, EE, ME, LE, ES [early stationary] and MS [mid stationary]), RNA was extracted from WT or Δ*5′tnpA* clones and processed for qRT‐PCR interrogating either *sopE* (A) or *hilD* (B) expression. The expression value for the WT strain in lag phase was set to 1. (C) HeLa invasion assay comparing invasion frequencies for the Δ*5′tnpA* strain versus the WT strain, harvested at EE or ES growth phase. The invasion frequency for the WT strain grown to EE phase was set to 1. Bars refer to the mean and error bars show the standard deviation. For A and B, *p* values (unpaired *T*‐test) and for C (One way ANOVA with Tukey's multiple comparison test) are indicated as follows: Ns, not statistically significant (*p* ≥ 0.05); *, **, *** and ****p ≤ 0.05, 0.01, 0.001 and 0.0001, respectively. For A, B, and C, 3, 6, and 5 independent clones were analyzed, respectively.

According to RNA‐seq, amongst the most highly upregulated genes in EE growth are those for the SPI‐1 effectors (e.g., *sopE2* [31‐fold], *sicA* [14‐fold], and *sipB* [13‐fold]) (Figure S4A). Thus, the increased level of expression of SPI‐1 TFs in EE growth was apparently sufficient to induce effector gene expression early in growth. A more detailed example of this is shown in the qRT‐PCR experiment shown in Figure 4A where we compare the expression profiles of *sopE* in WT and Δ*5′tnpA* strains over six growth points. A highlight from this experiment is the 35‐fold upregulation of *sopE* expression in the Δ*5′tnpA* strain compared to WT during early exponential (EE) growth. Quantification of *hilD* expression revealed a similar profile of increased levels in the Δ*5′tnpA* strain, supporting the notion that this increase was sufficient to drive effector expression early in growth (Figure 4B). Importantly, this increase in *hilD* and effector expression coincided with an increased invasion frequency for the Δ*5′tnpA* strain harvested at EE growth. As shown in Figure 4C, Δ*5′tnpA* cells grown to EE are close to 80‐fold more competent for invasion of HeLa cells versus WT bacteria taken at the same growth phase. In fact, the invasion frequency of the Δ*5′tnpA* strain taken at EE growth is roughly equivalent to that of the WT strain at early stationary phase, where SPI‐1 effector gene expression is maximal in LB media (Bustamante et al. 2008).

### 2.4 *5′* tnpA Modulates rtsA Expression in the HilD Auto‐Regulatory Loop, Potentially Through the sRNA PinT

**FIGURE 5:**
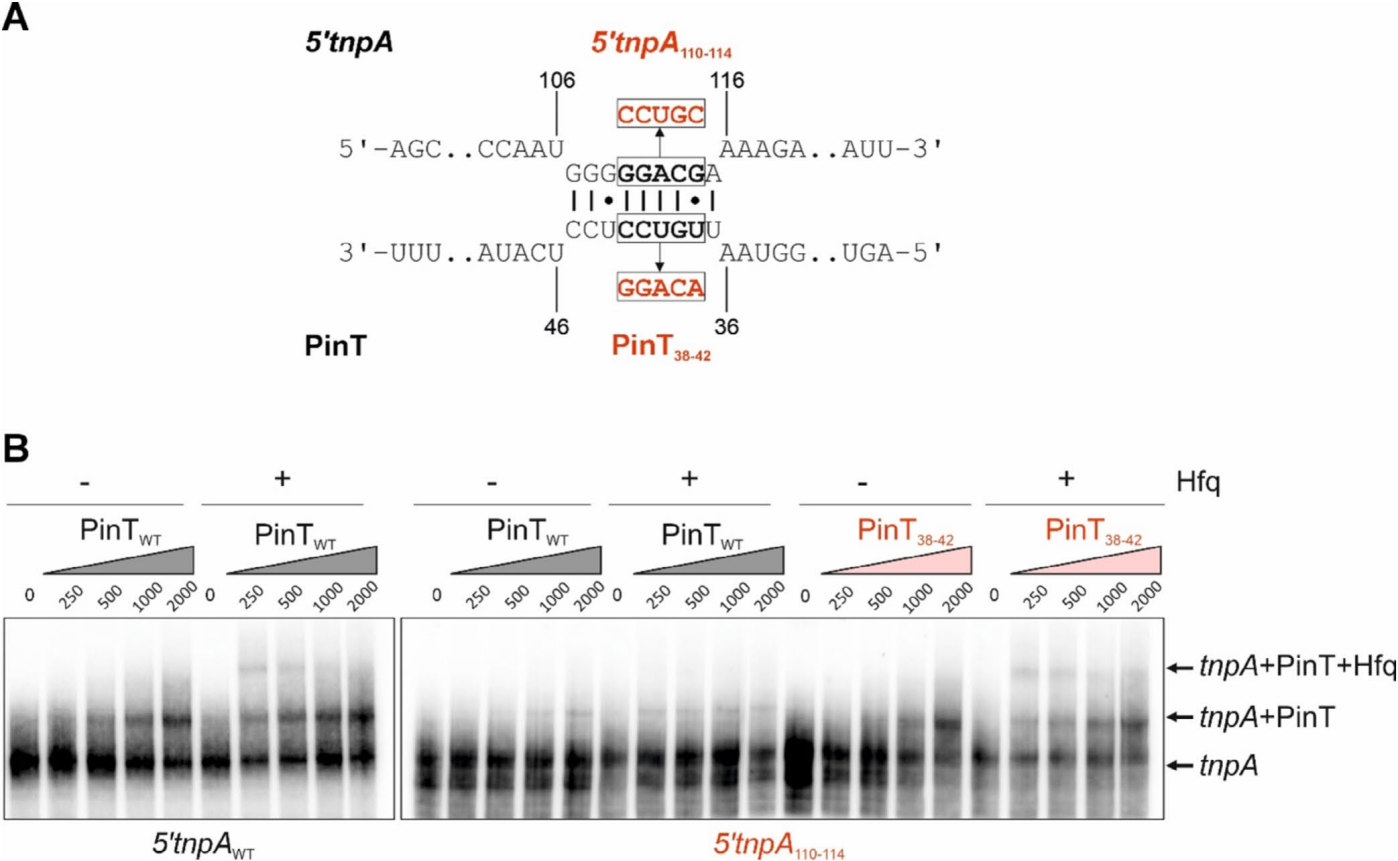
EMSA analysis confirms the interaction of *tnpA* with PinT in vitro. (A) Predicted binding site of *5′tnpA* and PinT sRNAs, as determined by IntaRNA. Mutated nucleobases are highlighted in red. (B) *5′tnpA* RNA (nt 1–260) was radioactively labeled, and an EMSA was performed to examine the binding interactions between PinT (0 nM; 250 nM; 500 nM; 1 mM; 2 mM) and the *tnpA* (4 nM) transcript in either the absence or presence of 100 nM Hfq. The left, middle and right panels show results for WT *5′tnpA* (*5′tnpA*_WT_) and WT PinT, mutant *5′tnpA* (*5′tnpA*_110–114_) and WT PinT, and mutant *5′tnpA*_110–114_ and compensatory mutant PinT (PinT_38–42_), respectively. Note that the *tnpA* transcript used here is a longer form than that generated in vivo (nt 1–125) through ribonucleolytic processing.

Results from the RNA‐seq analysis could hint at a mechanism of *5′tnpA*‐mediated regulation of SPI‐1. That is, the expression of *rtsA* was substantially more up‐regulated (8‐fold) in the Δ*5′tnpA* strain early in growth compared to *hilD* and *hilC* (just over 2‐fold) (Figure S4A). We validated this trend using qRT‐PCR analysis (Figure S7), suggesting that *5′tnpA* might act on an RtsA‐specific branch of the SPI‐1 regulatory pathway. Interestingly, previous work identified the sRNA PinT as a negative regulator of both *rtsA* and *hilA* at the posttranscriptional level (Kim et al. 2019). As PinT has previously been subjected to MS2 affinity purification and sequencing (MAPS) analysis (Correia Santos et al. 2021), we looked for evidence of a PinT‐*tnpA* interaction in these data sets. Indeed, the *5′*UTR of *tnpA* was found to be significantly enriched in the pulldown fraction of MS2‐tagged PinT as compared to the untagged PinT control (log2FC = 4.37; *p*‐adjusted = 0.02). *In silico* prediction for a possible pairing interaction between PinT and *tnpA* revealed substantial sequence complementarity between the two transcripts (Figure 5A). Notably, this putative PinT‐interaction site would fall ~87 nt downstream of the *5′tnpA* region known to base‐pair with *invF* mRNA (Ellis et al. 2017) (Figure 1, lower).

To test the putative interaction between PinT and *5′tnpA* experimentally, we performed electrophoretic mobility shift assays (EMSAs) on in vitro‐transcribed RNAs. Given that PinT is an Hfq‐dependent sRNA (Sittka et al. 2008) and *5′tnpA* has previously been shown to bind Hfq (Ellis et al. 2015), EMSAs were performed both in the presence and absence of this RNA chaperone. In these experiments, varying amounts of PinT were incubated with radio‐labeled *5′tnpA* (nt 1–260) prior to loading samples on a native polyacrylamide gel. The results in Figure 5B (left‐hand panel) show that even at the lowest concentration of PinT (250 nM) a substantial amount of the PinT‐5′tnpA duplex formed in the presence of Hfq (~30% of *5′tnpA* input was converted to complex). At higher concentrations of PinT, up to 50% of *5′tnpA* was converted to complex in the presence of Hfq. Pairing was strongly reduced—although not completely abolished—in the absence of Hfq, implying a strong dependence of the PinT‐5′tnpA interaction on Hfq.

### 2.5 In Vitro Interaction of *5′* tnpA With PinT and Ensuing SPI‐1 Repression Are Sequence‐Specific

To test the importance of the outlined regions of complementarity in complex formation, we mutated five potential pairing nucleotides in *5′tnpA* (hereafter referred to as *5′tnpA*_110–114_) and repeated the EMSA with wild‐type PinT. The results in Figure 5B (middle panel) show that complex formation was reduced in the presence of the *5′tnpA*_110–114_ mutant versus WT *5′tnpA* (maximum of 2% conversion to complex even in the presence of Hfq and at the highest concentration of input PinT). To confirm the importance of the base‐pairing interaction on complex formation, we introduced the compensatory mutations in PinT. The results in Figure 5B (right‐hand panel) show the rescue of complex formation with this mutant form of PinT and *5′tnpA*_110–114_. Like the fully WT reaction, the double mutant reaction gave about 30% conversion of input *5′tnpA* to complex in the presence of Hfq at the lowest input concentration of PinT. Based on these results and the previously obtained MAPS data (Correia Santos et al. 2021) we propose that *5′tnpA* and PinT sRNAs are binding partners, raising the possibility that *5′tnpA* impacts *rtsA* expression indirectly, through PinT.

**FIGURE 6:**
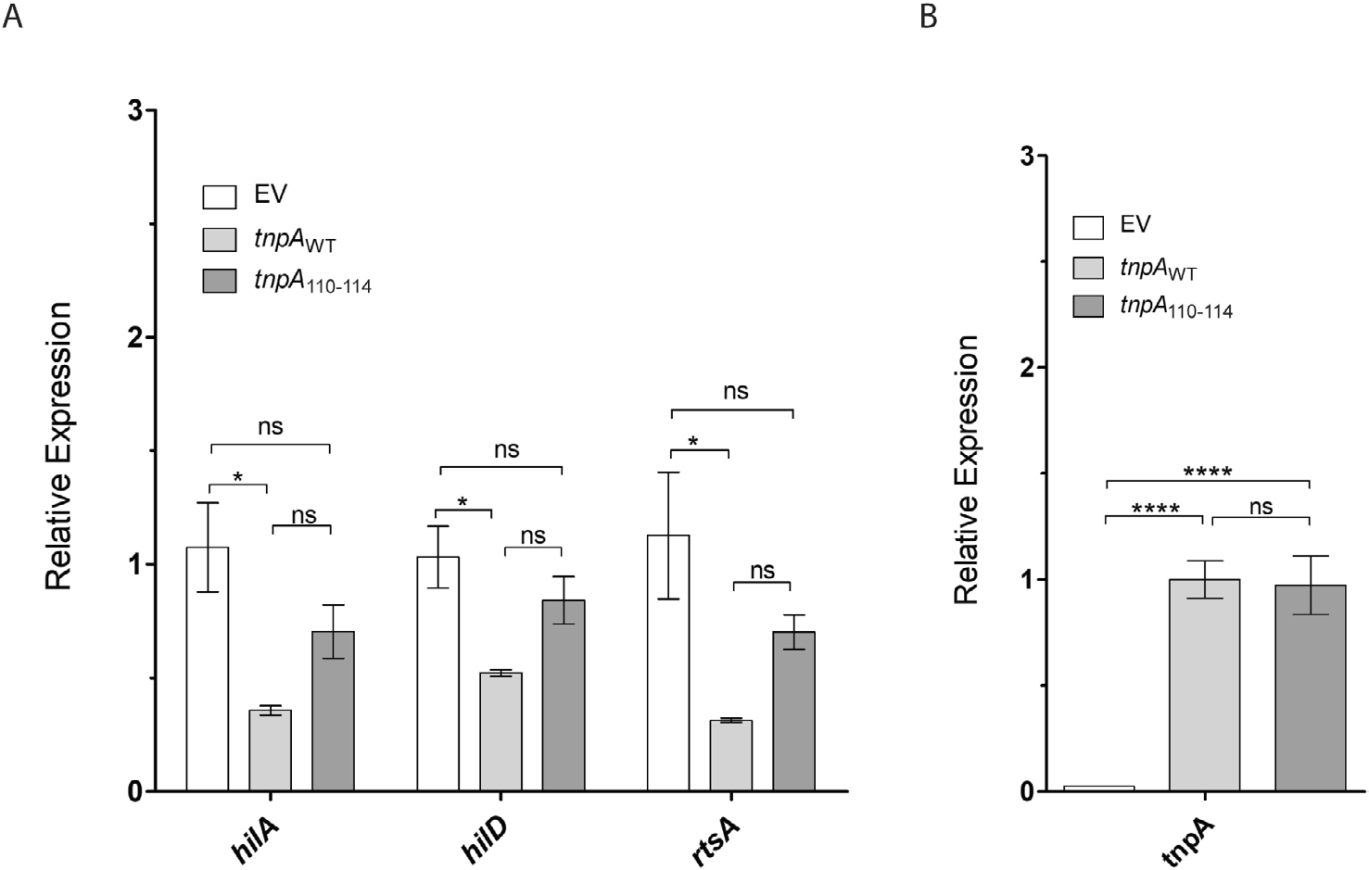
Impact of expression of WT and mutant forms of *tnpA* on select SPI‐1 transcription factor mRNAs. qRT‐PCR analysis of the Δ*5′tnpA* strain transformed with plasmids expressing either *tnpA*_WT_ or *tnpA*_110–114_, or harboring the empty vector (EV) control. In (A), the expression value for the EV‐containing strain was set to 1. In (B), the expression level of the *tnpA*_WT_‐expressing strain was set to 1. Bars refer to the mean and error bars show the standard deviation. *p* values (One way ANOVA with Tukey's multiple comparison test) are indicated as follows: Ns, not statistically significant (*p* ≥ 0.05); * and ****p ≤ 0.05 and 0.0001, respectively. Four independent clones were analyzed for each strain.

To further test this possibility, we introduced the 110–114 mutations into an IPTG‐inducible form of full‐length *tnpA* and compared the capacity of this (pDH1171) and the corresponding WT construct (pDH968) to suppress early induction of *rtsA*, *hilD*, and *hilA* expression in the Δ*5′tnpA* strain (Figure 6A). In this experiment, plasmids expressing either WT or mutant *tnpA* transcript, along with an empty vector (EV) control, were transformed into the Δ*5′tnpA* strain background. Cultures were grown in the presence of IPTG until EE phase and then processed for qRT‐PCR. The results show that WT *tnpA* expression strongly suppressed *rtsA*, *hilD*, and *hilA* expression (2‐ to 4‐fold). This supports our inference that the absence of *5′tnpA*–rather than the chimeric FLP‐scar‐3′tnpA transcript–was responsible for the early SPI‐1 induction phenotype observed in the Δ*5′tnpA* strain. Importantly, *tnpA*_110–114_ had a reduced capacity to suppress *rtsA*, *hilD*, and *hilA* expression, since there was no significant difference in any of these mRNA levels between the *tnpA*_110–114_ mutant and the EV control (Figure 6A). The nucleotide changes in the *tnpA*_110–114_ mutant did not influence the steady‐state level of this transcript (Figure 6B), arguing against the possibility that the reduced suppressive ability of this mutant was attributable to changes in transcript stability.

Lastly, we evaluated the degree to which *5′tnpA* was over‐expressed at 0.5 mM IPTG treatment relative to *5′tnpA* expression in the WT strain at EE growth. The results shown in Figure S8 indicate an approximate 3.5‐fold increase in *5′tnpA* transcript level in the over‐expression strain. In this experiment, we also titrated *5′tnpA* expression down to WT levels by reducing the IPTG concentration from 0.5 mM to 0.06 or 0.13 mM. At this reduced level of *5′tnpA* expression, we did not see evidence of *hilD* suppression (i.e., complementation was only observed at the higher *5′tnpA* expression level). While the significance of this finding is unclear, we note that lactose utilization genes are stochastically expressed (Ackermann 2015) and hence, bacterial responses to the synthetic lactose analog IPTG are inherently heterogenic (e.g., Binder et al. 2017). Thus, at the higher concentrations of IPTG, there may be a more uniform response to the inducer, whereas at the lower concentrations, only a subpopulation of cells may respond. Even if a threshold level of *5′tnpA* expression is reached in these responders, leading to *hilD* suppression, the unchanged *hilD* signal from non‐responding cells would mask this effect. Notably, the results presented in this section do not rule out the possibility that *5′tnpA* might act directly on *hilD*, *hilC*, or *rtsA* expression.

### 2.6 *5′* tnpA Also Regulates lrhA Expression to Influence SPI‐1 Induction

**FIGURE 7:**
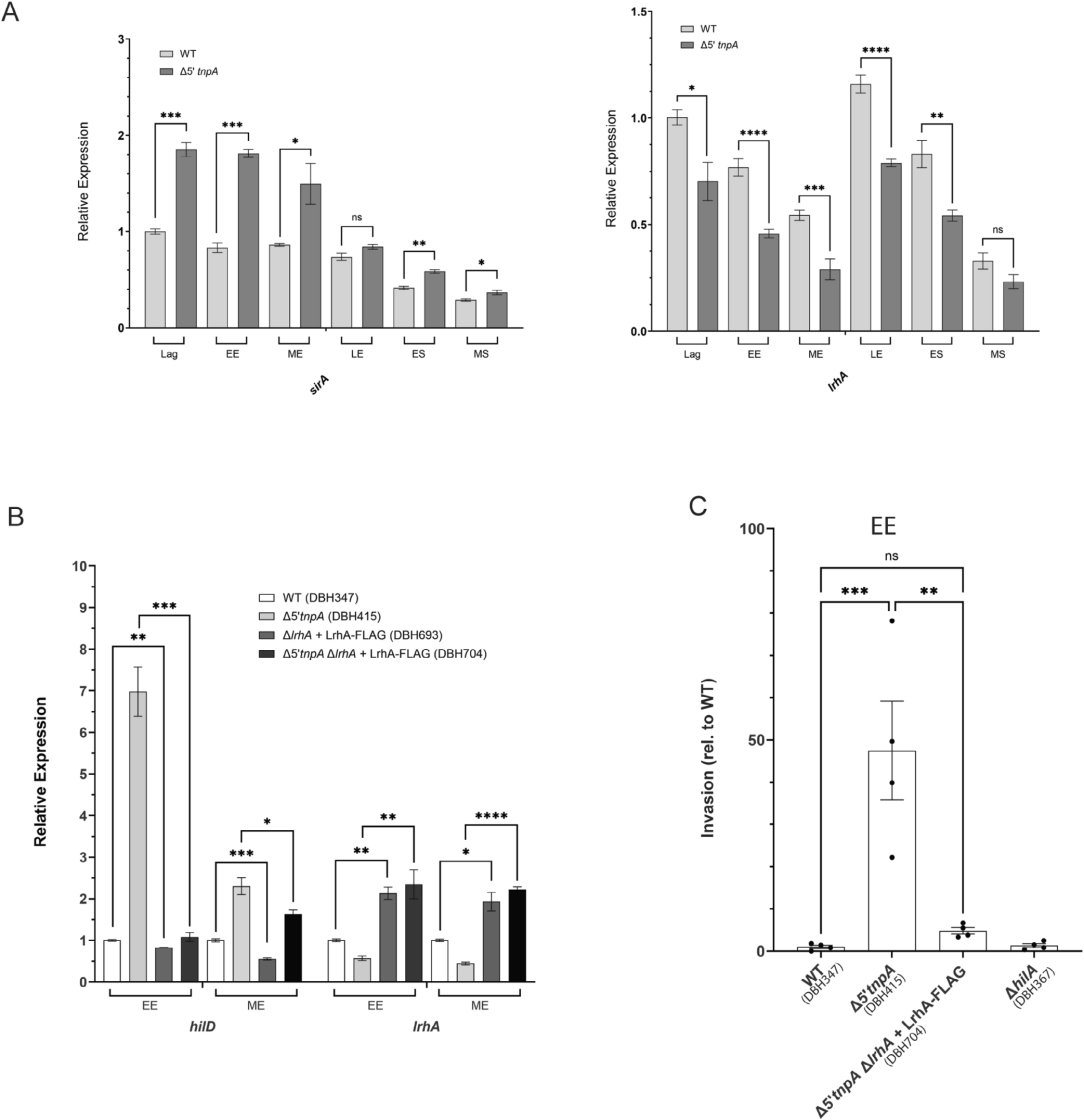
The Δ*5′tnpA* strain exhibits altered expression of *lrhA* and *sirA* early in growth. (A) *lrhA* and *sirA* expression profiles obtained through qRT‐PCR are shown for WT and Δ*5′tnpA* strains. (B) The impact of *lrhA* over‐expression on *hilD* expression as determined by qRT‐PCR. For each experiment in (A) and (B), the expression value for the WT strain in lag phase was set to 1. (C) The impact of *lrhA* over‐expression on *Salmonella* invasion of HeLa cells is shown. The invasion frequency for the WT strain was set to 1. Bars refer to the mean, and error bars show the standard deviation. *p* values (for A and B, unpaired *T*‐test; for C, One way ANOVA plus Tukey's multiple comparison test) are indicated as follows: Ns, not statistically significant (*p* ≥ 0.05); *, **, *** and ****p ≤ 0.05, 0.01, 0.001 and 0.0001, respectively. For A, B, and C, 6, 3, and 4 independent clones were analyzed, respectively.

The increase in *hilD* expression observed in the Δ*5′tnpA* strain might be entirely due to the impact of *5′tnpA* on *rtsA* (via PinT). Alternatively, *5′tnpA* might, in addition to *invF*, have other targets that regulate SPI‐1 expression. To assess this latter possibility, we asked if there was evidence of dysregulation of *hilD* regulators early in growth in the Δ*5′tnpA* strain. Note that evidence of this might not have been picked up by the RNA‐seq analysis if the extent of altered expression was relatively small (e.g., 2‐fold or less). Towards this end, we performed expression profiling via qRT‐PCR on several genes known to act upstream of *hilD* to regulate its expression, including *hilE* (Baxter et al. 2003), *ompR* (Cameron and Dorman 2012), *fur* (Troxell and Hassan 2013), *sirA* (Martínez et al. 2011), *rcsB* (Lin et al. 2008), and *lrhA*. The *lrhA* gene was included because it is known to be an early negative regulator of *flhDC* expression, which in turn controls the expression of the HilD regulator, FliZ (Mouslim and Hughes 2014). In addition, we had already established through RNA‐seq analysis that both *flhDC* and *fliZ* are upregulated early in growth (EE and ME) in the Δ*5′tnpA* strain (Figure S4A). Of the six genes profiled, only *sirA* and *lrhA* showed differential expression early in growth. The *sirA* gene was upregulated approximately 2‐fold starting from lag phase and continuing into ME phase, while *lrhA* exhibited reduced expression of about 2‐fold in EE and ME phases in the Δ*5′tnpA* strain (Figure 7A). In the current work, we focused on the differential expression of *lrhA*.

To assess the relative importance of reduced *lrhA* expression as it pertains to the early *hilD* induction phenotype characteristic of the Δ*5′tnpA* strain, we asked if over‐expression of *lrhA* in the Δ*5′tnpA* strain background has an impact on *hilD* expression and invasion. We were able to increase the *lrhA* transcript level by about 2‐fold by integrating a *lrhA‐FLAG* allele into the *attTn7* site of the *Salmonella* chromosome in a background where the native *lrhA* gene was disrupted (strains DBH693 and DBH704). We looked at the impact of this over‐expression on *hilD* transcript levels for cells grown to EE and ME growth phases. As shown in Figure 7B, increasing *lrhA* expression levels had little effect on *hilD* expression in an otherwise WT background (DBH693), but remarkably in the Δ*5′tnpA* strain background (DBH704) full suppression of the ‘*hilD*‐up’ phenotype was observed. Notably, this suppression was strongest for cells grown to EE phase. This fits with the idea that LrhA regulation of *flhDC*, and thus *fliZ*, is confined to EE growth (Mouslim and Hughes 2014). Invasion studies with the above strains also showed that *lrhA* over‐expression suppresses invasion to roughly WT levels early in growth (Figure 7C). Overall, the results here suggest that the reduced expression of *lrhA* in the Δ*5′tnpA* strain is a significant component of the early SPI‐1 induction phenotype. As there is not an established link between PinT and *lrhA* expression (Correia Santos et al. 2021), it is likely that the impact of *5′tnpA* on *lrhA* expression occurs through a pathway that does not involve PinT. Based on IntaRNA pairing predictions, there is a potential for a direct interaction between 5′*tnpA* and the *5′*UTR of the *lrhA* transcript (Figure S9). This comprises a *5′tnpA* sequence downstream of both the *invF* and PinT interaction sites (Figure 1), which should be tested in future experiments.

### 2.7 Coordinate Regulation of the Cysteine Regulon and phsABC Operon Through CysB

**FIGURE 8:**
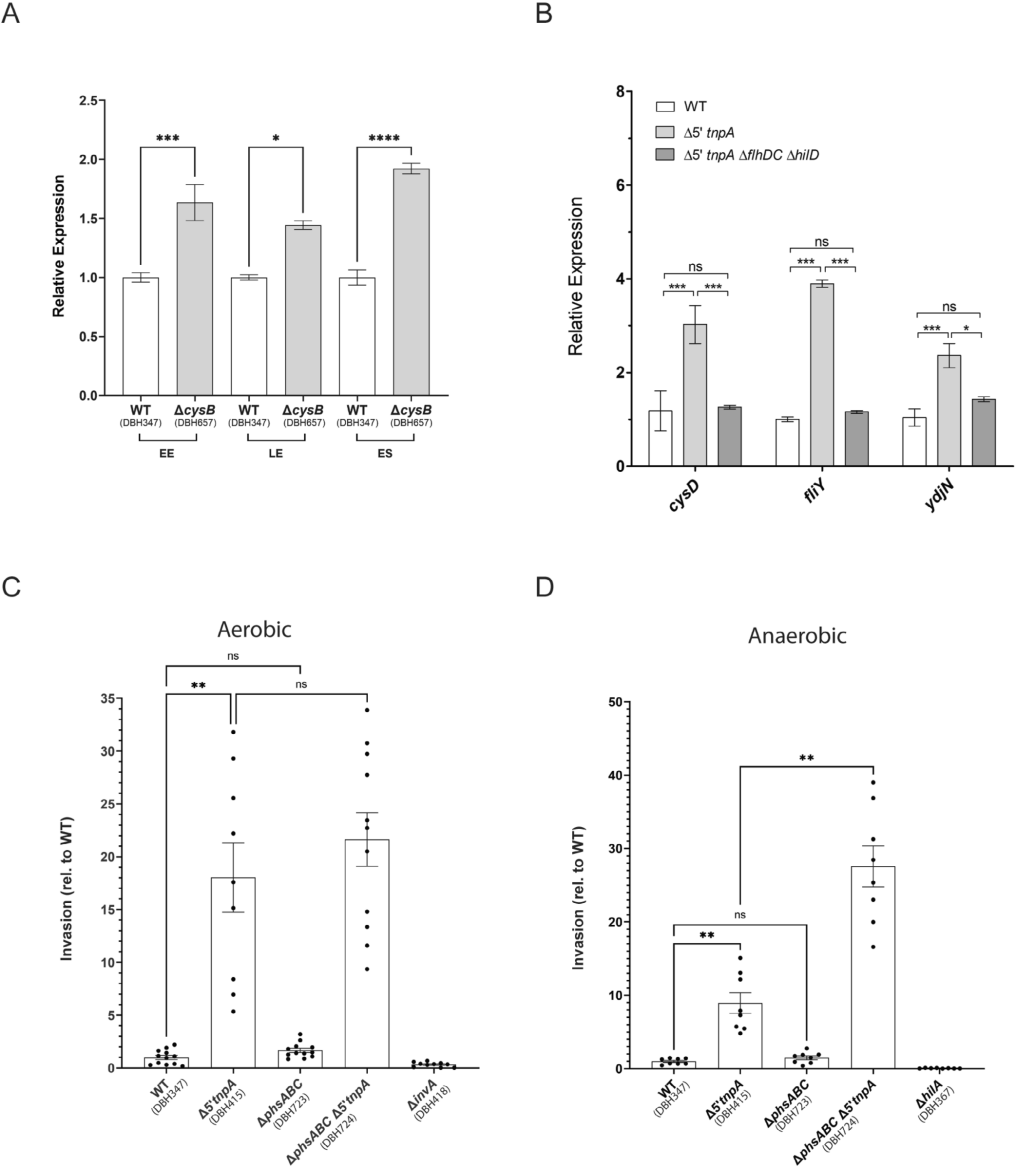
Impact of *cysB* deletion on *phsABC* expression and of *phsABC* deletion on *Salmonella* invasion. (A) Miller assay with *phsABC* translational fusion showing the impact of Δ*cysB* on *phsABC* expression. For each growth phase, the relative expression of *phsABC* was set to 1 for the WT strain. (B) The expression level of three cysteine regulon genes is compared through qRT‐PCR analysis for the indicated strains grown to LE phase. The expression of the target gene in WT in each group was set to 1. (C and D) Invasion assay performed under conditions where the *Salmonella* strains were grown either aerobically or anaerobically as indicated. The invasion frequency for the WT strain was set to 1. A noninvasive strain (either Δ*invA* or Δ*hilA*) served as a negative control. Bars refer to the mean and error bars show the standard deviation. *p* values (unpaired *T*‐test in A and B and One way ANOVA with Tukey's multiple comparison test in C and D) are indicated as follows: Ns, not statistically significant (*p* ≥ 0.05); *, **, *** and ****p ≤ 0.05, 0.01, 0.001 and 0.0001, respectively. For A, B, C, and D, 3, 4, 12, and 8 independent clones were analyzed, respectively.

Both the cysteine regulon and the *phsABC* operon influence cysteine biosynthesis, but in opposite ways. The cysteine regulon contributes by encoding proteins involved in sulfur acquisition (in the forms of thiosulfate, sulfate, and cystine) and its assimilation into cysteine (Kredich 1971; Baptist and Kredich 1977). In contrast, the thiosulfate reductase complex encoded by the *phsABC* operon diverts thiosulfate away from cysteine production (Clark and Barrett 1987). Given that the Δ*5′tnpA* strain shows opposing expression changes in these two gene sets, we hypothesized that their regulation might be mechanistically linked. Our working model is that early induction of invasion and flagellar regulons increases the normal demand for cysteine as part of metabolic stress. In response, this would trigger the upregulation of the cysteine regulon via activation of its master transcriptional regulator CysB (Kredich 1971) which would also repress *phsABC* operon expression. This regulatory mechanism aligns with the role of O‐acetyl serine, which accumulates under cysteine limitation and activates CysB (Kredich 1971). Scanning the *phsABC* operon for a potential CysB binding site revealed a reasonable match to the consensus sequence in between a previously identified Crp‐cAMP binding site and the −35 region of the *phsAB*C promoter (Figure S10). To test the idea that CysB regulates the *phsABC* operon, we looked at the impact of deleting *cysB* on the expression of a *phsABC‐lacZ* reporter (translational fusion). We show in Figure 8A that for cells in a WT background grown to EE, LE, and ES phase, disrupting the *cysB* gene consistently resulted in an increase in *phsABC* expression (up to a maximum of 2‐fold), consistent with CysB being a negative regulator of *phsABC* expression. We also looked at the dependence of cysteine regulon induction on SPI‐1 and flagellar gene expression. We show in Figure 8B that in the Δ*5′tnpA* strain background (DBH779) where SPI‐1 and flagellar gene expression was inhibited by deleting *hilD* and *flhDC* genes, respectively, the cysteine regulon induction typical for LE growth in the Δ*5′tnpA* strain did not occur.

Given the metabolic burden SPI‐1 and flagellar regulon expression place on the bacterial cell, we speculated that regulation of the *phsABC* operon might impinge on *Salmonella* invasion. That is, down‐regulation of the *phsABC* operon would increase cysteine availability, enhancing the production of proteins necessary for invasion and motility, thereby leading to a higher invasion frequency. Furthermore, this effect would only be observable in the Δ*5′tnpA* strain background, where early induction of SPI‐1 and flagellar regulons creates cysteine limitation. To determine if the *phsABC* expression has an impact on *Salmonella* invasion, we examined the consequences of its deletion. Initially, strains were grown under aerobic conditions for testing the efficacy of invading HeLa cells. Under these conditions, the *phsABC* disruption in either WT or Δ*5′tnpA* strain backgrounds did not have an impact on the invasion frequency (Figure 8C). However, since the thiosulfate reductase encoded by the *phsABC* operon is considered an anaerobic enzyme (Clark and Barrett 1987), we also monitored invasion frequencies under anaerobic growth conditions where the *phsABC* operon is more highly expressed (Figure S11A). Under anaerobic growth conditions with cells in ES phase, Δ*phsABC* increased the invasion frequency approximately 3‐fold (Figure 8D). Notably, this increase was only observed when the *phsABC* operon was disrupted in the Δ*5′tnpA* strain background. We also isolated RNA from the anaerobically growing strains used for invasion for qRT‐PCR analysis. This analysis revealed that the increase in invasion observed for the Δ*5′tnpA*Δ*phsABC* strain (DBH724) relative to the Δ*5′tnpA* strain (DBH415) is not due to differences in *hilD* expression levels (Figure S11B). These results support the idea that maintaining a critical threshold level of cysteine production is important, specifically under anaerobic growth conditions, for ensuring adequate expression levels of SPI‐1 and/or flagellar proteins needed to fully support *Salmonella* invasion.

## 3 Discussion

**FIGURE 9:**
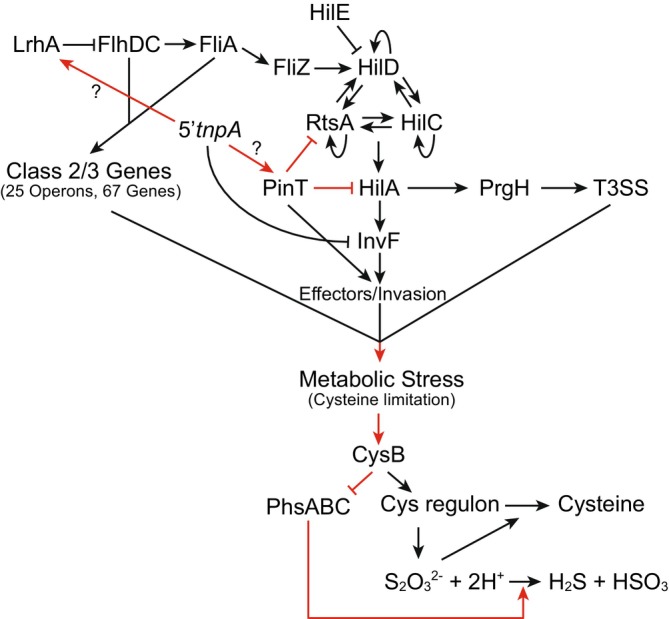
Model for *5′tnpA*‐mediated regulations of SPI‐1, flagellar, cysteine and *phsABC* regulons. The *5′tnpA* sRNA is known to be a direct negative regulator of *invF* expression (Ellis et al. 2017) and we here propose it to also interact with PinT, thereby positively impacting on PinT's activity through an unknown mechanism. Additionally, *5′tnpA* may directly interact with, and regulate *lrhA*. In the absence of *5′tnpA* (as in the Δ*5′tnpA* strain), PinT function is expected to decrease with a resultant increase in both *rtsA* and *hilA* expression (which are established negative targets of this sRNA (Kim et al. 2019)). Increased levels of RtsA early in growth are predicted to jump‐start the HilD positive feedback loop. The decrease in *lrhA* expression in the Δ*5′tnpA* strain early in growth may account for the increase in *flhDC*, *fliA* and *fliZ* expression, the latter of which also promotes HilD function due to the posttranslational effect of FliZ on HilD (Cott Chubiz et al. 2010). Increased early expression of SPI‐1 and the flagellar regulons is predicted to impact on both the expression of cysteine and *phsABC* regulons. This is thought to occur through increased CysB function, a consequence of cysteine shortage associated with the metabolic stress resulting from early induction of these large regulons. In this capacity, CysB is a positive regulator of the cysteine regulon and a negative regulator of the *phsABC* operon. Reduced expression of the *phsABC* operon would increase the amount of thiosulfate (S_2_O_3_^2−^) in the media available for assimilation into cysteine. Red lines indicate new pathways revealed or proposed by the current work. Note also that this is a simplified version of the SPI‐1 regulatory pathway; for a detailed version of the SPI‐1 regulatory cascade, see (Ellermeier and Slauch 2007).

In the current work, we investigated the impact of deleting the 5′ portion of the IS200 *tnpA* transcript (*5′tnpA*) on the transcriptome of the pathogenic *Salmonella* strain SL1344 in rich media. Comparative RNA‐seq identified over 200 differentially expressed genes throughout exponential growth. While these changes represent the combined effects of both direct and indirect mechanisms of this sRNA, the results are consistent with *5′tnpA* being a regulatory component of multiple *Salmonella* gene expression networks (Figure 9). Importantly, the increased expression of SPI‐1 and flagellar regulon genes, which occurred early in growth in the Δ*5′tnpA* strain, likely contributed to this strain having an increased capacity to invade HeLa cells, indicating that the observed gene expression changes are functionally significant. Although based primarily on correlation, we present data that suggest that these effects may be partially explained by *5′tnpA* acting as a direct regulator of the sRNA PinT, which was previously shown to be a key regulator of the host cell invasion regulon (Westermann et al. 2016; Kim et al. 2019; Correia Santos et al. 2021; Kooshapour et al. 2025). Additionally, *5′tnpA* may bind and regulate *lrhA*, encoding a flagellar protein cross‐talking with SPI‐1 genes, yet validation of *lrhA* mRNA as a direct *5′tnpA* target is pending. We also identified genes involved in sulfur metabolism (cysteine regulon and the *phsABC* operon) as being coordinately regulated in the Δ*5′tnpA* strain. We suggest that this is an indirect effect of deletion of *5′tnpA* arising due to metabolic stress caused by the early induction of invasion and flagellar regulons causing cysteine shortage.

### 3.1 Scope of Transposon‐Encoded sRNA Regulation of Bacterial Host Gene Expression

Despite their threat to genome integrity, transposons are ubiquitous in genomes in all three kingdoms of life. It is thought that the successful spread and retention of transposons in genomes reflects their ability to evolve defenses against host mechanisms aimed at suppressing transposon activity, and in some cases providing functions that can contribute to host fitness (Cosby et al. 2019). In bacteria, one example of the latter is that transposons have the capacity to acquire and disseminate antibiotic resistance genes to their hosts. Another example is the domestication of transposon‐encoded Cas proteins to function in host defense against invading phages (i.e., the evolution of CRISPR defense systems) (Chylinski et al. 2014; Krupovic et al. 2014; Kapitonov et al. 2016). In this and previous work (Ellis et al. 2017), we have provided evidence for IS200 domestication in *Salmonella*. However, unlike the Cas example, in the case of IS200 it is the function of a transposon‐derived sRNA rather than a protein that has been exapted by the host.

Is the integration of *5′tnpA* into *Salmonella* regulatory circuits an isolated example of transposon domestication? IS200 is the founding member of the IS200/IS605 family of insertion sequences. This family is made up of three groups (the IS200, IS605, and IS1341 groups) and has approximately 153 members (Siguier et al. 2015). A common feature within the IS200 group, which includes insertion sequences that are widespread in enteric bacteria including *Salmonella*, *E. coli* , *Shigella*, and *Yersinia*, is the presence of an inverted repeat in the *5′*UTR of the *tnpA* transcript. In *Salmonella*, it has been shown that the 5′ portion of the transcript contains a stable stem‐loop structure that sequesters the ribosome‐binding site, thereby inhibiting translation of the *tnpA* coding sequence (Beuzón and Casadesús 1997; Beuzón et al. 2004). This is one of several mechanisms that have evolved to reduce TnpA expression and to keep IS200 transposition exceedingly low, minimizing negative selection against the transposon (Ellis et al. 2015). We propose here that this structural feature of the *tnpA* transcript can benefit the host by giving rise to a stable sRNA that is available for posttranscriptional regulatory functions. Furthermore, if targets of this sRNA include regulatory molecules that are conserved in enteric bacteria (e.g., regulators of the flagellar regulon such as *lrhA*), one could anticipate that the example of domestication reported here will be widespread. However, as the copy number of IS200 group insertion sequences is typically quite high, alternate methods of silencing these elements to gene disruption (used here) such as CRISPR interference (Larson et al. 2013; Prezza et al. 2024) would likely need to be applied to uncover such examples. Interestingly, another example of an IS element‐encoded sRNA impacting host function has recently been reported. In this case, a remnant of a *tnpA* gene present in IS3000, a Tn3 family member, was shown to express an sRNA in response to Fosfomycin stress that directly interacts with a host protein, YadG, to modulate the efflux of this antibiotic (Lin et al. 2025).

### 3.2 Linking *Salmonella*'s Early Invasion Phenotype to the Absence of *5′tnpA*

The generation of the Δ*5′tnpA* strain involved multiple genetic manipulations (7 independent P22 transductions and four rounds of flipping out antibiotic resistance genes used for selection in the transductions). In addition to the removal of the first 261 nt of the *tnpA* transcript from all seven copies of IS200 in the SL1344 genome, our genetic manipulation generated new transcription units in which the *FRT‐scar* sequence is fused to the last 409 nt of *tnpA* in seven locations of the genome. It was therefore important to ensure that the loss of the *5′tnpA* sequence, rather than the creation of a novel transcript and/or altered transcription at one or more of the modified sites, was responsible for the novel properties of the Δ*5′tnpA* strain. Regarding the latter possibility, one of the disruptions (*tnpA4*) is immediately upstream of the *fliA* gene (Figure S1), which encodes *σ* ^28^ that drives the expression of *fliZ* (Ikebe et al. 1999). Accordingly, if the disruption of *tnpA4* were to increase expression of *fliA*, this would increase FliZ levels, which in turn would increase HilD activity and SPI‐1 induction. We tested the impact of this disruption alone in an otherwise WT background on *fliZ* and *hilD* expression via qRT‐PCR and found no increases in expression of either of these genes (Figure S6). Further support that the early SPI‐1 induction phenotype characteristic of the Δ*5′tnpA* strain is due to loss of the 5′ portion of *tnpA* comes from our *tnpA* over‐expression experiment where we showed that over‐expression of full‐length *tnpA* suppressed the increase in the expression of mRNAs for select SPI‐1 TFs (HilD, HilA, and RtsA) that is characteristic of this mutant strain. One possible reason that suppression of *hilD* and downstream factors was limited to approximately 2‐fold may be that this construct also produces the antisense RNA *art200*, which pairs with the *tnpA* transcript promoting its degradation (Ellis et al. 2015).

### 3.3 Plausible Mechanisms of *5′tnpA* 's Regulatory Function

Small regulatory RNAs in bacteria can function by multiple mechanisms (Storz et al. 2011; Hör et al. 2020). Some specialized sRNAs can titrate RNA‐binding proteins, preventing these proteins from binding other transcripts (Lawhon et al. 2003; Romeo and Babitzke 2018). The vast majority, however, function through complementary base‐pairing with target RNAs, influencing the translation of the target RNA and/or its stability (Hör et al. 2020). In Gram‐negative bacteria, the pairing interaction is typically catalyzed by an RNA‐binding chaperone, either Hfq or ProQ (Vogel and Luisi 2011; Olejniczak and Storz 2017; Holmqvist and Vogel 2018), which provides a nucleation center for the RNA‐pairing partners. As an emerging theme, sRNAs can also act as decoys titrating other sRNAs, through an RNA–RNA pairing mechanism, so that they are no longer available to pair with a partner mRNA (Mandin and Gottesman 2009; Figueroa‐Bossi and Bossi 2018; Denham 2020).

With regard to understanding how *tnpA* functions as a regulator of the SPI‐1 regulon, we thought it potentially significant that the absence of *5′tnpA* had a much stronger impact on the expression of only one component of the *hilD‐hilC‐rtsA* positive feedback loop, namely *rtsA*. Given that *rtsA* had previously been shown to be negatively regulated by the sRNA PinT (Kim et al. 2019), we looked for evidence of an interaction between *tnpA* and PinT. *In silico* prediction of interaction sites and reanalysis of existing MAPS data supported this hypothesis, and EMSA experiments validated *5′tnpA*‐PinT base‐pairing in the presence of Hfq in vitro. Importantly, introducing specific point mutations to abolish *5′tnpA*‐PinT interaction in the *Salmonella* chromosome suggested this duplex formation could be important for several of the observed *5′tnpA*‐dependent expression changes. Interestingly, rather than one sRNA acting as a sponge for the other, our data imply that *5′tnpA* bolsters PinT‐mediated target repression of RtsA and HilA (Figure 9), which would be a relatively unusual mechanism that warrants further investigation.

It is interesting to note that PinT harbors two known seed regions: the first one, located at position ~7–23 nt, mediates base‐pairing to the *rtsA* and *hilA* mRNAs (Kim et al. 2019), whereas the second (~27–43 nt) engages with its other known target mRNAs (Westermann et al. 2016; Correia Santos et al. 2021) and—as we showed here (Figure 5)—interacts with *5′tnpA* in vitro. It is therefore conceivable that *5′tnpA* binding may selectively prevent PinT from interacting with target mRNAs other than *rtsA* and *hilA*, thereby fostering regulation of the latter through reduced competition. Notably, the sRNA InvS—that is, the latest addition to the list of PinT targets (Kooshapour et al. 2025) is essential for *Salmonella* invasion into HeLa cells (Wang et al. 2017). Hence, *5′tnpA* may not only foster PinT‐mediated repression of target mRNAs, but also PinT‐mediated sponging of InvS, together contributing to the hyper‐invasive phenotype of Δ*5′tnpA* mutants in epithelial cell culture. Finally, while PinT steady‐state levels remained largely unaffected by the deletion of *5′tnpA* over growth in rich medium (Figure S4A), this does not formally rule out an impact of *5′tnpA* on PinT's half‐life. For example, *5′tnpA* binding might occlude a sequence otherwise accessible for endonucleolytic cleavage.

For sRNAs that function through complementary pairing with target RNAs, it is not unusual to have multiple targets (Papenfort and Vogel 2010). This may be the case for *5′tnpA*, where we have previously shown that this sRNA targets the *invF* mRNA and now propose that it could also target PinT and the *lrhA* transcript. The (predicted) interaction sites for these three targets do not overlap, as shown in Figure 1, implying binding to different targets may not necessarily be mutually exclusive. Notably, both the *invF* and PinT interaction sites are present within the first 120 nucleotides of the *5′tnpA* transcript, which is liberated from the full‐length transcript by endoribonucleolytic processing (Ellis et al. 2017). This portion of the transcript appears to be the most stable part of the transcript and includes a long (~100 nt) hairpin loop structure (Figure S12). The *invF* pairing region is in the mostly unstructured 5′ portion of the 5′tnpA transcript, and the putative PinT pairing region is in the mostly unstructured 3′ portion of this processed RNA. A further processing event by ribonuclease E at nt 19 (Chao et al. 2017) has the potential to dismantle the *invF* pairing site, hinting at another layer of regulation. The potential *lrhA* pairing site within the *5′tnpA* transcript is downstream of the processing site, immediately adjacent to the PinT pairing sequence, and consequently is not present in the most stable form of the *tnpA* transcript. Accordingly, regulation of *lrhA* by *5′tnpA* would rely on the presence of a population of either unprocessed transcript or a processed form where only the extreme *5′* end of the transcript is removed.

Collectively, our data do not exclude the possibility that *5′tnpA* can function through the titration of one or more RNA‐binding proteins. At this point, we have only established that the *5′* portion of *tnpA* is capable of binding Hfq (Ellis et al. 2015). Other RNA‐binding proteins that would be worth investigating for a *5′tnpA* transcript interaction include CsrA, ProQ, and CspC/E, as these proteins have been implicated in SPI‐1 and/or flagellar regulon gene expression (Lawhon et al. 2003; Michaux et al. 2017; Potts et al. 2019; Westermann et al. 2019). In fact, the minimal binding site for CsrA (*5′*GGA) (Holmqvist et al. 2016), CspC or CspE (*5′*CUG and *5′*CAG, respectively) (Yair et al. 2022) are present in 3, 3, and 4 copies, respectively, within the *5′tnpA* sequence, and ProQ has been shown to bind *art200* (Smirnov et al. 2016) and therefore likely plays some role in establishing *5′tnpA* transcript levels.

### 3.4 An Indirect Linkage Between *5′tnpA* and the Coordinate Regulation of the Cysteine Regulon and the *phsABC* Operon

We found it curious that the expression of two systems functioning in sulfur metabolism was differentially expressed in opposite directions in the Δ*5′tnpA* strain in LE growth. We suggest that the observed upregulation of the cysteine regulon and the downregulation of the *phsABC* operon are coupled responses to metabolic stress associated with the early induction of invasion and flagellar regulons. Notably, we demonstrated that cysteine regulon induction did not occur in LE growth in the Δ*5′tnpA* strain when SPI‐1 and flagellar gene expression was blocked through deleting both *hilD* and *flhDC* genes. Mechanistically, how might changes in cysteine regulon and *phsABC* operon genes be coordinated? The cysteine regulon is induced when cysteine concentrations are low (Ostrowski and Kredich 1989). Under these conditions, L‐serine is converted to O‐acetylserine (OAS) by the enzyme CysE, which is inhibited by cysteine. OAS and its alternate conformer N‐acetylserine bind to the TF CysB, allowing CysB to bind DNA and activate downstream transcription in a sequence‐specific manner (Kredich 1992). We suggested that induction of invasion and flagellar genes would lead to a cysteine shortage and thus induction of the cysteine regulon. One possible way of coordinating cysteine regulon and *phsABC* operon expression might then be through CysB. In support of this possibility, we demonstrated that in a strain where the *cysB* gene was disrupted, *phsABC* expression increased about 2‐fold, consistent with CysB acting as a negative regulator of *phsABC* expression (Figure 9).

Based on the above findings, we postulated that deletion of the *phsABC* operon would positively impact invasion because this deletion would increase sulfur flux towards cysteine (and methionine) production, thereby better allowing cells to cope with metabolic stress arising from SPI‐1 and flagellar regulon induction. In fact, deletion of the *phsABC* operon did increase invasion about 3‐fold. This increase was specific to the Δ*5′tnpA* strain under anaerobic growth conditions. The dependence of this response on the absence of *5′tnpA* fits with the notion that increased invasion is dependent on the metabolic stress linked to induction of invasion and flagellar regulons. Overall, these results are consistent with the idea that downregulating *phsABC* operon expression provides a means for cells to recover from the metabolic stress associated with SPI‐1 and flagellar regulon induction.

In summary, this study revealed a transposon‐derived sRNA to act as a global regulator of *Salmonella* invasion and flagellar genes. In addition to dysregulation of these regulons through the deletion of *5′tnpA* sRNA, the resultant early induction phenotype appears to also drive dysregulation of cysteine biosynthesis, presumably due to the metabolic stress associated with the early induction of these large regulons. Given that regulatory RNAs have become an increasingly important target for therapeutic strategies (Zhao et al. 2022), we suggest that the IS200 *5′tnpA* could represent an attractive drug target for *Salmonella* anti‐infective therapy.

## 4 Experimental Procedures

### 4.1 Growth Conditions, Strains and Plasmids

For experiments performed under both aerobic and anaerobic conditions, *S*. Typhimurium was grown at 37°C in Lennox Broth (LB; 5 g/L NaCl, 10 g/L tryptone, 5 g/L yeast extract) supplemented with Streptomycin (150 μg/mL). For aerobic growth, standard culture tubes (16 × 150 mm) were used and cultures were grown with shaking. For anaerobic growth, cultures were grown without shaking in 2 mL microtubes (Axygen) filled to the top with LB. After approximately 24 h, anaerobic cultures reached an OD_600_ ≅ 0.5; the culture density did not change significantly with further incubation time, indicating that the cultures had reached stationary phase. For all aerobic experiments, overnight cultures were diluted 1:100 and then grown to the desired density (see points on growth curve in Figure S3). For experiments where strains were transformed with plasmids, plasmids were maintained by selection with ampicillin or kanamycin at 200 μg/mL and 50 μg/mL, respectively.

All strains and plasmids used in this study are listed in Table S5, and oligonucleotides are listed in Table S6. *S* Typhimurium str. SL1344 was considered the WT strain, and derivatives were made in the SL1344 background. *Escherichia coli* DH5α was used for routine cloning and plasmid propagation, and *E. coli* MG1655 and SL1344 genomic DNA were used as templates for PCR amplification where indicated.

Mutant strains of SL1344 were constructed by Lambda Red recombineering (Datsenko and Wanner 2000), P22 HT105/1 *int‐201* (P22)‐mediated transduction (Maloy et al. 1996) or where indicated mini‐Tn7‐mediated transposition of genes into the *attTn7* site of the *Salmonella* chromosome (Shivak et al. 2016) (see Supporting Information for details). Colony PCR was used to confirm all new constructs, and where indicated, antibiotic resistance cassettes were removed using the temperature‐sensitive plasmid pCP20 (pDH739) carrying the FLP recombinase (Cherepanov and Wackernagel 1995).

Each of the disrupted copies of IS200 in the Δ*5′tnpA* strain retains the weak *tnpA* promoter, the *tnpA* transcription start site, an *FRT*‐scar sequence, and internal *tnpA* sequence extending from bp 301 (nt 261) to bp 710 (nt 670) (Figure 1). We made the disruption in this way to minimize the impact of each IS200 disruption on surrounding genes. The location of each of the seven disrupted copies of IS200, including immediately surrounding transcription units, is shown in Figure S1. Based on the raw reads from the RNA‐seq analysis, a form of *tnpA* for each of the disrupted IS200 copies in the Δ*5′tnpA* strain is expressed (Table S1). In principle, this transcript should be a chimera wherein the *FRT*‐scar sequence is fused to a portion of the *tnpA* transcript consisting of nt 261–670.

### 4.2 RNA Isolation

Total RNA was prepared by the hot acid phenol method (Aiba et al. 1981) and quantified using a nano‐drop spectrophotometer (IMPLEN).

### 4.3 RNA‐Seq and Data Analysis

Three colonies of each of WT and *Δ5′tnpA* strains were grown to EE, ME and LE phases whereupon 600 μL were removed and processed for RNA extraction. Purified RNA was treated with TURBO DNase (Ambion) to remove residual genomic DNA and ~500 ng of total, DNase‐treated RNA was treated with the Ribo‐Zero “Bacteria” (Illumina), following the manufacturer's instructions. rRNA‐depleted RNA was precipitated in ethanol for 3 h at −20°C. cDNA libraries for Illumina sequencing were generated by Vertis Biotechnologie AG, Freising‐Weihenstephan, Germany. To this end, rRNA‐free RNA samples were first sheared via ultrasound sonication (4 pulses of 30 s at 4°C) to generate on average 200–400 nt fragments. Fragments < 20 nt were removed using the Agencourt RNAClean XP kit (Beckman Coulter Genomics) and the Illumina TruSeq adapter was ligated to the 3′ end of the remaining fragments. First‐strand cDNA synthesis was performed using M‐MLV reverse transcriptase (NEB) wherein the 3′ adapter served as a primer. The first‐strand cDNA was purified and the *5′* Illumina TruSeq sequencing adapter was ligated to the 3′ end of the antisense cDNA. The resulting cDNA was PCR‐amplified to about 10–20 ng/μL using a high fidelity DNA polymerase. The TruSeq barcode sequences were part of the 5′ and 3′ TruSeq sequencing adapters. The cDNA libraries were purified using the Agencourt AMPure XP kit (Beckman Coulter Genomics) and analyzed by capillary electrophoresis (Shimadzu MultiNA microchip). Prior to sequencing, individual cDNA samples were pooled in equimolar amounts. The resulting cDNA pool was size‐fractionated in the range of 200–600 bp using a differential clean‐up with the Agencourt AMPure kit (Beckman Coulter Genomics). Aliquots of the cDNA pools were analyzed by capillary electrophoresis (Shimadzu MultiNA microchip). Sequencing was performed on a NextSeq 500 platform (Illumina) at Vertis Biotechnologie AG, Freising‐Weihenstephan, Germany (single‐end mode; 75 cycles). All RNA‐seq data discussed in this publication is available upon request.

Reads were aligned to the *S. typhimurium* SL1344 genome (NC_016810.1) with Rockhopper (McClure et al. 2013) (Table S1) and differential expression was analyzed using ALDEx2 (Fernandes et al. 2014). More detail on data analysis is provided in Supporting Information.

### 4.4 Reverse Transcription Quantitative Polymerase Chain Reaction (qRT‐PCR)

DNase treated RNA (2 μg) was converted to cDNA with the High‐Capacity cDNA Reverse Transcription Kit (Applied Biosystems); cDNA was diluted to 30 ng/μL in TE [50 mM Tris–HCl, pH 8.0, 1 mM Ethyleneediaminetetraacteticacid (EDTA)] and stored at −20°C. A minimum of three biological replicates were analyzed in technical duplicate in each experiment, and the 16S rRNA (*rrsA*) was used as a reference gene for relative quantitation. Reactions (48 μL) contained 360 ng of cDNA, 500 nM of each primer (Table S6) and PowerUP SYBR Green Master Mix (Applied Biosystems). Standard settings on the ViiA 7 Real‐Time PCR system were used except for the anneal/extension step, which was performed at 60.5°C. Relative expression of each target was calculated by the efficiency corrected method (Pfaffl 2001).

### 4.5 Growth Curves

Growth was measured in a Multiskan Go microplate spectrophotometer. Cells from 3 overnight cultures (biological triplicates) of each strain (WT and Δ*5′tnpA*) were diluted 100‐fold in LB. 200 μL of each dilution was plated in technical replicates in a 96‐well microplate. Cultures were grown with continuous shaking at 37°C for 12 h, and absorbance at 600 nm (A_600_) was measured every 15 min. Note that the A_600_ was not adjusted for path length and light scattering from the microplate lid and is therefore not directly comparable to optical density readings measured in a standard cuvette. Absorbance readings from the plate reader were calibrated to a spectrophotometer, allowing us to use cuvette readings to identify cultures at specific growth phases for RNA extraction.

### 4.6 β‐Galactosidase Assays

Overnight cultures transformed with the *phsABC* reporter plasmid (pDH1120) were grown in LB supplemented with ampicillin (200 μg/mL) and then diluted 1:100 and grown to the indicated growth phase. The Miller assay was performed as previously described (Ross et al. 2010).

### 4.7 Gentamicin Protection (Invasion) Assay

Tissue culture plates (12‐well) were seeded with ~0.05 X 10^6^ HeLa cells per well in 1 mL of Dulbecco's Modified Eagle's Medium (DMEM) supplemented with 10% (v/v) fetal bovine serum (FBS) and kanamycin (300 μg/mL) 20–22 h prior to the invasion assay. At the time of the assay, cells were 70%–80% confluent (~0.1 × 10^6^ cells per well). For invasion assays performed with aerobically and anaerobically grown strains, strains were grown as previously described from freshly streaked colonies. Bacterial cells were washed with 0.85% NaCl saline solution and resuspended in DMEM/10% FBS to a concentration of 1 × 10^7^ cfu/mL with a final volume of 1 mL.

HeLa cells were washed with Dulbecco's Phosphate Buffered Saline (dPBS) and 1 mL of bacterial suspension (MOI of 40) was added per well. Serial dilutions of the bacterial suspension were plated in technical duplicates on LB agar plates supplemented with 150 μg/mL streptomycin to determine the input number of bacteria.

Bacterial cells were centrifuged onto the HeLa cell monolayer at 100 *X g* for 3 min at room temperature and then incubated at 37°C for 40 min. Bacterial cells were washed with dPBS, which was then followed by the addition of 100 μg/mL gentamicin in DMEM/10% FBS for 1.5 h incubation at 37°C to kill extracellular bacteria. After washing with dPBS, HeLa cells were resuspended in 250 μL of lysis solution (0.1% Triton X‐100 in 1X dPBS) and serial dilutions were plated in technical duplicates on LB with 150 μg/mL of streptomycin to determine the output bacterial cell counts. Invasion was calculated as the ratio of recovered cells to the input and normalized to the indicated strain.

### 4.8 *In Silico* Prediction of Complementary Regions Between *5′tnpA* and Potential Targets (PinT and *lrhA* )

*5′tnpA*‐PinT and *5′tnpA‐lrhA* interactions were predicted using Freiburg RNA tools‐IntaRNA (Mann et al. 2017). Regarding the *5′tnpA*‐PinT interaction, mutated bases are highlighted in red in Figure 5, with nucleotide positions annotated relative to the *5′* end (+1) of the sRNAs and start codon of the mRNA. The predicted hybridization energy was −7.64 kcal/mol. For the *5′tnpA‐lrhA* interaction, the predicted hybridization energy was—4.67 kcal/mol.

### 4.9 In Vitro Transcription and RNA Labeling

A 200 ng DNA fragment, PCR‐amplified from *Salmonella* genomic DNA, was used as a template for transcription with the MEGAscript T7 Transcription Kit (Thermo Fisher Scientific). The RNA's size and integrity were verified on a denaturing polyacrylamide gel. RNA bands were excised, eluted overnight at 4°C in RNA elution buffer (0.1 M sodium acetate, 0.1% SDS, 10 mM EDTA), extracted with phenol:chloroform:isoamyl alcohol (P:C:I), and precipitated with ethanol. For dephosphorylation, 50 pmol of RNA was treated with 10 units of calf intestinal phosphatase (CIP, New England Biolabs) at 37°C for 1 h in a 50 μL reaction, followed by P:C:I extraction and ethanol precipitation. Subsequently, 20 pmol of the dephosphorylated RNA was *5′*‐labeled with 32P‐γ‐ATP (10 μCi/μL) using T4 polynucleotide kinase (Thermo Fisher Scientific) in a 20 μL reaction at 37°C for 1 h. Unincorporated nucleotides were removed using microspin G‐50 columns (GE Healthcare) as per the manufacturer's protocol.

### 4.10 Electrophoretic Mobility Shift Assay

Each reaction contained 0.04 pmol of radiolabeled RNA, which was denatured at 95°C for 1 min, chilled on ice for 5 min, and renatured in 1X structure buffer (10 mM Tris–HCl pH 7.0, 0.1 M KCl, 10 mM MgCl₂) at 37°C for 10 min. For each reaction, 1 μg of yeast RNA was included, and labeled RNA was mixed with increasing concentrations of unlabeled RNA. Where applicable, 100 nM of purified Hfq was added to the reaction. Reactions were incubated at 37°C for 15 min, then stopped with 5X RNA native loading buffer. Samples were separated on 6% native polyacrylamide gels at 4°C in 0.5X TBE buffer using a constant current of 40 mA for 3–4 h. Gels were dried, signals detected with a Typhoon FLA 7000 phosphoimager, and images visualized using EMBL ImageJ software.

## Author Contributions

**Ryan S. Trussler:** investigation, writing – review and editing, formal analysis. **Naomi‐Jean Q. Scherba:** investigation, writing – review and editing, formal analysis. **Hoda Kooshapour:** investigation, writing – review and editing, formal analysis. **Michael J. Ellis:** software, formal analysis. **Konrad U. Förstner:** data curation, software, formal analysis, methodology, investigation. **Matthew Albert:** investigation, writing – review and editing. **Alexander J. Westermann:** investigation, methodology, validation, writing – review and editing, supervision, formal analysis. **David B. Haniford:** supervision, conceptualization, investigation, funding acquisition, writing – original draft, writing – review and editing, project administration.

## Supporting information


Data S1.

## Data Availability

The data that support the findings of this study are available on request from the corresponding author. The data are not publicly available due to privacy or ethical restrictions.
