# Supplementary material for: A Small RNA Derived From the 5′ End of the IS200 tnpA Transcript Regulates Multiple Virulence Regulons in Salmonella typhimurium
Source: Mol Microbiol. 2025 Aug 13;124(5):413–32. doi: 10.1111/mmi.70016 (PMC12594629; doi:10.1111/mmi.70016)
Supplement: Supplementary file 1 — Data S1. [file MMI-124-413-s001.zip › mmi70016-sup-0001-DataS1.docx]

**SUPPLEMENTARY INFORMATION**

A small RNA derived from the 5’ end of the IS200 *tnpA* transcript regulates multiple virulence pathways in *Salmonella* Typhimurium

Ryan S. Trussler^1^, Naomi-Jean Q. Scherba^1^, Hoda Kooshapour^2,3^, Michael J. Ellis^4^, Konrad Forstner^5^, Matthew Albert^1^, Alexander J. Westermann^2,3#^ and David B. Haniford^1#^

^1^Department of Biochemistry, University of Western Ontario, London, Ontario, Canada N6A 5C1

^2^ZB MED, Information Centre for Life Sciences, Cologne, Germany

^3^Department of Microbiology, Biocenter, University of Würzburg, Würzburg, Germany

^4^BenchSci, Toronto, Ontario, Canada M6G 1A9

^5^Helmholtz Institute for RNA-based Infection Research (HIRI), Helmholtz Centre for Infection Research (HZI), Würzburg, Germany

^#^Co-corresponding author: Email [haniford@uwo.ca](mailto:haniford@uwo.ca); Tel (+1) 519-661-4013; [alexander.westermann@uni-wuerzburg.de](mailto:alexander.westermann@uni-wuerzburg.de); Tel (+49) 931-31-83781

**Supplementary Experimental Procedures**

**Strain and plasmid construction**

Strains DBH367, 468, 554, 555, 657, 693, 704, 722, 723, 724 and 779 listed in Table S5 were generated by P22 transduction of either WT or Δ*5’tnpA* strains (Maloy *et al.*, 1996). Transducing lysates containing the corresponding disruption alleles used to construct the above strains were generated from strains obtained from other laboratories or generated by the Haniford lab via lambda red recombineering (Datsenko and Wanner, 2000). Strain DBH689 was generated using the modified Tn7 transposition system (Shivak *et al.*, 2016). Briefly, the *lrhA-3X-FLAG* was cloned into pDH1004, a derivative of pUC18R6K-mini-Tn7-*PacI* (pDH1001) containing a *kan^R^* gene, producing pDH1100. This new plasmid was transformed by electroporation into the WT strain (DBH347) containing pDH1002 (a source of Tn7 transposase). After a 2 hr recovery at 30°C cells with Tn7 hops were selected for by plating on LB Kan plates at 37°C. Colony PCR was used to detect the presence of the *lrhA-3X-FLAG* gene at the *attTn7* site of the *Salmonella* chromosome. DBH689 was then used to produce a P22 transducing lysate for transducing the *attTn7-lrhA-3X-FLAG*-*kan^R^* cassette into DBH554 and DBH555. *Δ5’tnpA* was constructed as previously described (Ellis *et al.*, 2017).

Plasmids expressing *tnpA* under the control of the IPTG-inducible PLlacO-1 promoter, including the full-length gene (pDH968) and the *tnpA_110-114_* mutant (pDH1171), were constructed as follows. For pDH968 a PCR product encoding the full length *tnpA* gene was amplified from DBH347 genomic DNA with oDH747 and oDH753, digested with *AatII* + *MfeI* and cloned into an *AatII* + *EcoRI* digested derivative of pBR-plac (pDH763). This places the *tnpA* gene under the control of the pLlacO-1 promoter. The *lacI^q^* gene was also cloned into this backbone at a unique *HindIII* site. For pDH1171 construction a modified SOEing technique was used. In the initial PCR with Q5 polymerase oligonucleotides oDH860 (mutagenic oligonucleotide) and oDH748 (*tnpA*-specific primer) were used with pDH968 as template to produce PCR product 1 consisting of approximately 100 bp of the ‘front end’ of the *tnpA* gene. In the second PCR, gel purified PCR product 1 and oDH861 (*tnpA*-specific primer) were used as primers with pDH968 as template and GO Taq polymerase to generate PCR product 2 (~ 450 bp) consisting of most of the *tnpA* gene. After gel purification, PCR product 2 was digested with *AatII* and *EcoR1* and cloned into the pDH968 backbone also digested with *AatII* and *EcoR1*. The *phsA-lacZ* translational fusion (pDH1120) was constructed by first cloning a PCR product containing a portion of the *phsA* gene (including codons 1-47) into *EcoRI-PstI* digested pBR322. The PCR product was amplified from SL1344 with oDH847 and oDH848 and digested with *EcoRI* and *PstI*. Then the *lacZ* gene (starting at codon 11) present on a *PstI* digest product was cloned into the *PstI* site creating pDH1120. For hopping genes into the *attTn7* site of the SL1344 chromosome we first generated a *mini-Tn7* containing construct (pDH1004) with the *kan^R^* gene from IS903 situated between the Tn7 end sequences. For this the *kan^R^* gene flanked by *PsiI* sites was amplified using oDH841 and oDH842 and the PCR product was cut with *PsiI* and cloned in *HinCII* digested pDH1001. Subsequently, *lrhA-3X-FLAG* (pDH1100), was introduced into pDH1004 as follows. We first fused the *3X-FLAG* sequence to the C-terminus of the *lrhA* gene by generating a PCR fragment with SL1344 genomic DNA as template and primers oDH845 and oDH846. The corresponding PCR product was digested with *EcoRI* and *HindIII* and cloned into *EcoRI-HindIII* cut pDH1093 (a derivative of pDH337 in which the *lacZ-alpha* promoter was removed via *HinCII/PshAI* digest) producing pDH1101. This *lrhA-3X-FLAG* sequence was then purified after *SacI-NruI* digestion and cloned into *SacI-EcoRV* cut pDH1004 generating pDH1100. A plasmid expressing the *ruvB* gene under the control of the pBAD promoter (pDH1078) was constructed by cloning a PCR product generated with primers oDH862 and oDH863 and SL1344 genomic DNA into pBAD24 cut with *EcoRI* and *HindIII*.

**RNA-seq Data Analysis**

Transcripts predicted by Rockhopper (McClure *et al.*, 2013) were manually annotated with the genomic interval and categorized into intergenic (predicted RNA, e.g. 4824999-4825049_predicted RNA) or intragenic (antisense, e.g. 1906043-1906092_antisense: fadR) RNAs prior to expression analysis (Table S1). Differential expression was analyzed using ALDEx2 version 1.3.4.0 a tool for analyzing the compositional nature of high-throughput sequencing data (Fernandes *et al.*, 2014). Since ALDEx2 compares two conditions, this analysis was performed on each pairwise comparison (i.e. WT EE vs *Δ5’tnpA* EE, WT ME vs *Δ5’tnpA* ME, WT LE vs *Δ5’tnpA* LE) (Table S2). Briefly, the relative expression (abundance) of each gene within a sample was calculated as the median centered log-ratio (clr) from 1000 Monte-Carlo Dirichlet instances. The ALDEx2 effect size was then calculated for each feature as the median log2 difference between conditions (i.e. strains) divided by the maximum difference within a condition (i.e. difference in relative abundance between biological replicates). Accordingly, the effect size is a measure of the confidence one has in the difference in expression for a gene between two conditions, but does not necessarily inform on the magnitude of the difference. An effect size of 2 means that the difference between conditions (i.e. log2 fold-change between two strains) is twice as large as the greater difference within a condition (variance between biological replicates). We took a more conservative approach for considering genes differentially expressed and used an effect size of 2 as the cutoff for differential expression (Table S3).

**SUPPLEMENTAL TABLES**

**Table S1.** All RNA-seq reads mapped to the *S.* Typhimurium SL1344 genome using Rockhopper. Each pairwise comparison is shown on a separate sheet in Excel (Sheet 1, WT EE vs Δ*5’tnpA* EE; Sheet 2, WT ME vs Δ*5’tnpA* ME; Sheet 3, WT LE vs Δ*5’tnpA* LE).

**Table S2**. Results of ALDEx2 analysis of RNA-seq data. Each pairwise comparison (Sheet 1, WT EE vs Δ*5’tnpA* EE; Sheet 2, WT ME vs Δ*5’tnpA* ME; Sheet 3, WT LE vs Δ*5’tnpA* LE) was made using ALDEx2.

**Table S3**. Genes identified as differentially expressed (|Effect| >2) in our RNA-seq experiment. Each pairwise comparison is shown on a separate sheet in Excel (Sheet 1, WT EE vs Δ*5’tnpA* EE; Sheet 2, WT ME vs Δ*5’tnpA* ME; Sheet 3, WT LE vs Δ*5’tnpA* LE).

**Table S4.** Groupings of differentially expressed genes from Venn diagram. Lists of differentially expressed genes found in 1, 2 or 3 different growth phases depicted in the Venn diagram of Figure 2F. The total number of differentially expressed genes in each category is indicated.

**Table S5. Bacterial strains and plasmids used in this study.**

|  | Relevant genotype/ description | Reference/source |
| --- | --- | --- |
| *S*. Typhimurium |  |  |
| DBH199 | Wild-type LT2, Str^R^ | Miguel Valvano |
| DBH347 | Wild-type SL1344, Str^R^ | Salmonella Genetic Stock Centre (SGSC438) |
| DBH367 | DBH347 Δ*hilA::kan^R^*, Kan^R^Str^R^ | This study |
| DBH415 | DBH347 Δ*tnpA_2::FRT* Δ*tnpA_7::FRT* Δ*tnpA_6::FRT* Δ*tnpA_4::FRT* Δ*tnpA_1::FRT* Δ*tnpA_5::FRT* Δ*tnpA_3::FRT*, Str^R^ | (Ellis *et al.*, 2017) |
| DBH418 | SL1344 Δ*invA*, Str^R^ | Brian Coombes |
| DBH428 | DBH199 *ΔhilD::kan^R^*, Kan^R^ | This study |
| DBH458 | JS1155 *ΔflhDC::cm^R^*, Cm^R^Str^R^ | James Slauch |
| DBH468 | DBH415 *ΔflhDC::cm^R^*, Cm^R^Str^R^ | This study |
| DBH554 | DBH347 Δ*lrhA::kan^R^*, Kan^R^Str^R^ | This study |
| DBH555 | DBH415 Δ*lrhA::kan^R^*, Kan^R^Str^R^ | This study |
| DBH657 | DBH347 Δ*cysB::kan^R^*, Kan^R^Str^R^ | This study |
| DBH693 | DBH347 Δ*lrhA::FRT attTn7-lrhA-3X-FLAG-kan^R^*, Kan^R^Str^R^ | This study |
| DBH704 | DBH415 Δ*lrhA::FRT attTn7-lrhA-3X-FLAG-kan^R^*, Kan^R^Str^R^ | This study |
| DBH722 | DBH199 Δ*phsABC::kan^R^*, Kan^R^Str^R^ | This study |
| DBH723 | DBH347 Δ*phsABC::kan^R^*, Kan^R^Str^R^ | This study |
| DBH724 | DBH415 Δ*phsABC::kan^R^*, Kan^R^Str^R^ | This study |
| DBH779 | DBH415 *ΔflhDC::cm^R^* *ΔhilD::kan^R^*, Cm^R^Kan^R^Str^R^ | This study |
|  |  |  |
| Plasmids |  |  |
| pDH337 | pWKS30, low copy plasmid with multiple cloning sites, *pSC101 ori* and Ap^R^ | (Fu Wang and Kushner, 1991) |
| pDH739 | pCP20, temperature sensitive FLP expression plasmid, Ap^R^Cm^R^ | (Datsenko and Wanner, 2000) |
| pDH740 | pKD46, temperature sensitive Lamda Red expression plasmid, Ap^R^ | (Datsenko and Wanner, 2000) |
| pDH742 | pKD4, kanamycin cassette for Lambda Red recombineering, Ap^R^Kn^R^ | (Datsenko and Wanner, 2000) |
| pDH763 | pBR322 with *pLlacO-1* promoter and multiple cloning sites, Ap^R^ | Susan Gottesman |
| pDH859 | pBAD24 | ATTC |
| pDH968 | pBR322-derived plasmid expressing *tnpA_Full-Length_* from the *pLlacO-1* promoter and LacI^q^, Ap^R^ | This study |
| pDH1001 | pUC18*R6K*-*mini-Tn7-PacI*, Ap^R^ | Aaron White |
| pDH1002 | pHSG415-t*nsABCD*, Ap^R^ pSC101*ori^ts^* | Aaron White |
| pDH1004 | pDH1001 *mini-Tn7-kan^R^*, Ap^R^ Kan^R^ | This study |
| pDH1078 | pBAD24 containing the *ruvB* gene, Ap^R^ | This study |
| pDH1093 | pWKS30 *ΔplacZ*, Ap^R^ | This study |
| pDH1100 | pDH1004 *mini-Tn7-kan^R^-lrhA-3X-FLAG*, Ap^R^Kan^R^ | This study |
| pDH1101 | pDH1093 with *lrhA-3X FLAG*, Ap^R^Kan^R^ | This study |
| pDH1120 | pBR322-derived *phsA-lacZ* translational fusion, Tet^R^ | This study |
| pDH1171 | pDH968 with *tnpA_110-114_* mutations, Ap^R^ | This study |
| pSS048 | PinT plasmid with compensatory mutations to *tnpA_110-114_*, Ap^R^ | Jorg Vogel |

**Table S6. Oligonucleotides used in this study.**

| Name | Sequence (5’ to 3’) | Description/use |
| --- | --- | --- |
| Strain and Plasmid Construction | | |
| oDH841 | TATGTCTTATAACAACAAAGCCACGTTGTGTCTC | Amplifying Kan gene with PsiI cut sites |
| oDH842 | TATGTCTTATAAGTTACAACCAATTAACCAATTCTG |  |
| oDH845 | TTACGAATTCTCATCATTAGTGTCGCGTCG | Amplifying native lrhA with C-term 3xFLAG |
| oDH846 | ACTAAGCTTTTACTATTTATCGTCGTCATCTTTGTAGTCGATATCATGATCTTTATAATCACCGTCATGGTCTTTGTAGTCCTCAAAACCCCCTTCCACC |  |
| oDH747 | TACCGAGACGTCTCAGCTTTAAGCCAGTTATTAAA | Amplifying full length tnpA for inducible expression |
| oDH753 | ATCTAACAATTGTAAATATCCTCCGGCATAGCC |  |
| oDH847 | TTACGAATTCCTTATCAACATCATTA | Amplifying phsA TLF fragment |
| oDH848 | TTACTGCAGCAAGCTTGGCGTTAGCGTG |  |
|  |  |  |
| oDH860 | GGTGTGCGCTAAGCTCTTTTGCAGGCCCATTGGGACCCCCTTTTG | Amplifying *tnpA* to generate 110-114 mutant |
| oDH861 | AACTGCTCGTAAAGCATCAGACTAC |  |
| oDH862 | AGGAGGAATTCACCATGATAGAAGCAGATCGCCTG | Amplifying ruvB for insertion into pBAD24 |
| oDH863 | CCCAAGCTTCTAAGGCATCTCTGGCGG |  |
| AWO-2095 | GTTTTTTTAATACGACTCACTATAGGAGCTTTCAGCTTTAAGC | Amplifying tnpA fused to T7 promoter for IVT |
| AWO-2097 | AATTCGTACGTTTTTCCAT |  |
| AWO-2140 | GGTCCCAATGGGCCTGCAAAAGAGCTT | Amplifying *tnpA_110-115_* mutant for IVT |
| AWO-2141 | AAGCTCTTTTGCAGGCCCATTGGGACC |  |
| JVO-20898 | GTTTTTTAATACGACTCACTATAGGGGTAACGGATTACTTTGTGG | Amplifying PinT fused to T7 promoter for IVT |
| JVO-9436 | AAAAAAGCGGCAGACTACGC |  |
|  |  |  |
| qRT-PCR | | |
| oDH704 | TTCAGTTGGCATACTGCCGC | *sicA* |
| oDH705 | GAAGGCGCCACGCTAAAAGA |  |
| oDH815 | GCTGTGTTTCGCTTTCCAGC | *flhDC* |
| oDH816 | TTCGCGCGTCGATGACTTAC |  |
| oDH804 | AGGTAATTGCCAAGGCGTCG | *fliZ* |
| oDH805 | CTACTTGACGACGCTGCCTG |  |
| oDH661 | TCTGGGAAACTGCCTGATGGAGG | *rrsA* (16S rRNA) |
| oDH662 | CGTAGGAGTCTGGACCGTGTCTC |  |
| oDH794 | TGCCGGTGACCATTACGAAG | *hilA* |
| oDH795 | AGAGAGAAGCGGGTTGGTGT |  |
| oDH710 | GGTCTCCTGATACTGGTGCG | *invF* |
| oDH711 | AGCCCGGAAGCATGGTTTAT |  |
| oDH394 | TATTTGGGCGCGAAAACTATG | *tnpA* |
| oDH428 | GTTGCCAGACCGCAAGATG |  |
| oDH764 | GCCAGCTGCGGATAATAGGT | *prgH* |
| oDH765 | TGTCGCTGCGCAAAATGAAA |  |
| oDH788 | CGTCCTGCCGCAGATAACTT | *hilD* |
| oDH789 | TGAGCACCAACATCCCAGGT |  |
| oDH817 | CCTGTGACCATTTGCGGGTG | *hilC* |
| oDH818 | TGGCATGTCCACGGGTTTG |  |
| oDH819 | AGCCAGACCCGTGAAGCTAT | *sopE* |
| oDH820 | TAAAAGGATTGGCCCCTGCG |  |
| oDH798 | GCAACTGGAGGCGGAAAGTA | *cysD* |
| oDH799 | CGCGAAAGGCGTACATCTCA |  |
| oDH800 | TGAGCAACAATGGGCGAAGG | *cysP* |
| oDH801 | GTTGTTCGGCAGACGGCTTT |  |
| oDH821 | TGTTCCCGGAAACCTACCAGT | *cysH* |
| oDH822 | TTCAGCTCTTTTAGCGCCCG |  |
| oDH823 | GCGGCGCTATCGATGATTGT | *cysM* |
| oDH824 | AATCAGTTCCGCGCCATAGG |  |
| oDH802 | GCAGGCCCGTCATAAACAGT | *ydjN* |
| oDH803 | CGAATACCAGCGGCATCACA |  |
| oDH778 | GAGCTTTGCCGATGAGGGTT | *fliY* |
| oDH779 | CAGCATTCCGTCCCATTTGGT |  |
| oDH784 | TCTGCTGCTGGGATTTGTGC | *yecS* |
| oDH785 | GCCGCGGTATTGAGCGATAA |  |
| oDH825 | AAAAGTGGGGCGTGGGATTC | *sbp* |
| oDH826 | GTCGCCTGTTTACCCGAACC |  |
| oDH827 | AACCTATGAAACGCACCGGG | *phsA* |
| oDH828 | ATTAGGCGAACCAAAGGCCG |  |
| oDH829 | GTCGAGCGGGCAATTTTACG | *sirA* |
| oDH830 | TGATGACCACGAACTGGTGC |  |
| oDH835 | TCAGTCAGCAAATGCAGCGT | *lrhA* |
| oDH836 | CTGATTCATCAGAAGCGCCGA |  |

**Supplementary Figure Legends**

**
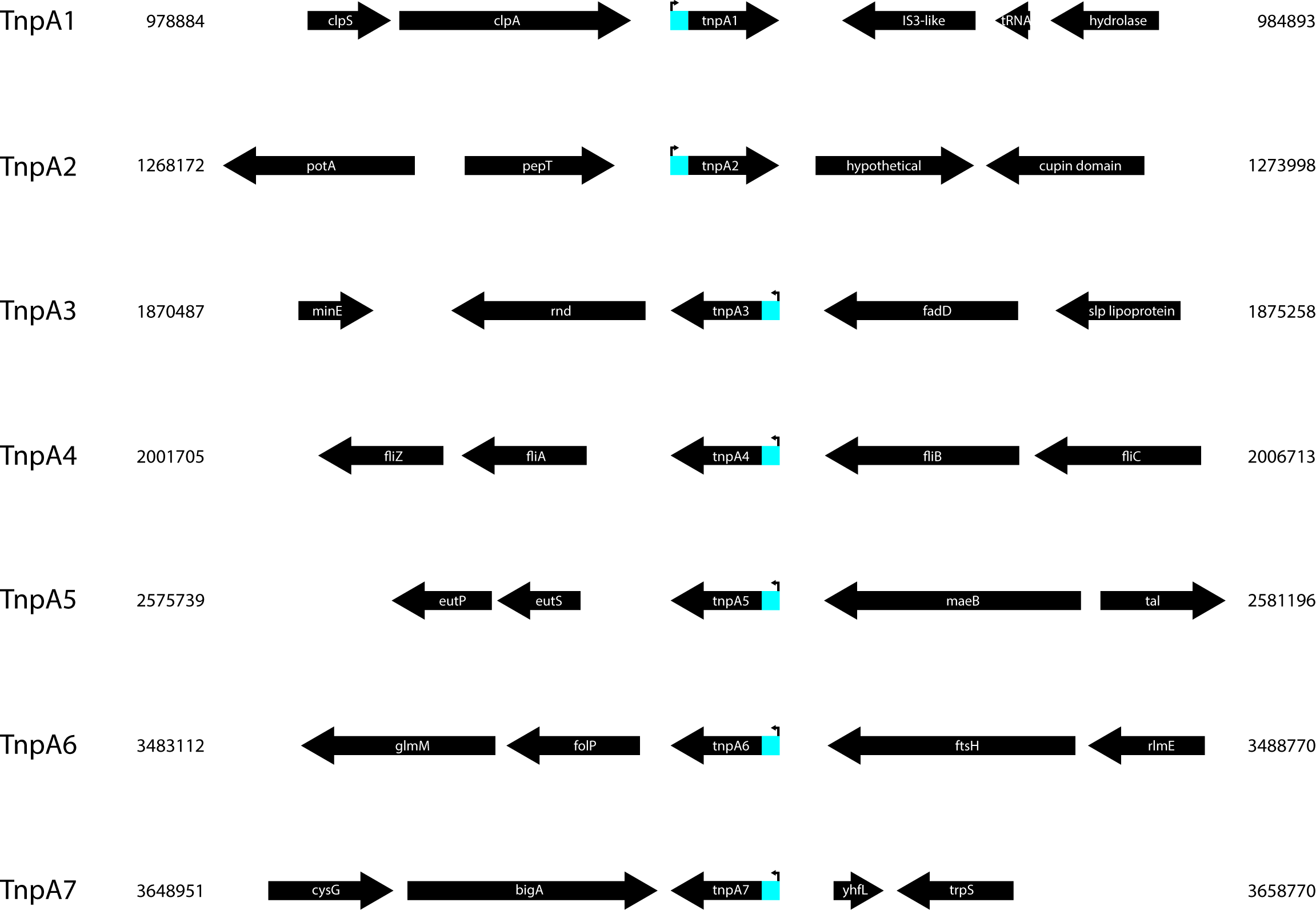
**

**Figure S1.** The position of all 7 disrupted copies of IS200 in the *Salmonella* SL1344 genome is shown. Arrowheads define the orientation of each disrupted IS200 element along with the direction of transcription of immediately neighboring genes. The small arrow defines the IS200 *tnpA* promoter. The blue rectangle is the *FRT-scar* sequence generated by flipping out antibiotic resistance genes used for selection in the construction of the *Δ5’tnpA* strain. Numbers define the genetic interval where each copy of IS200 is located within the *Salmonella* chromosome.


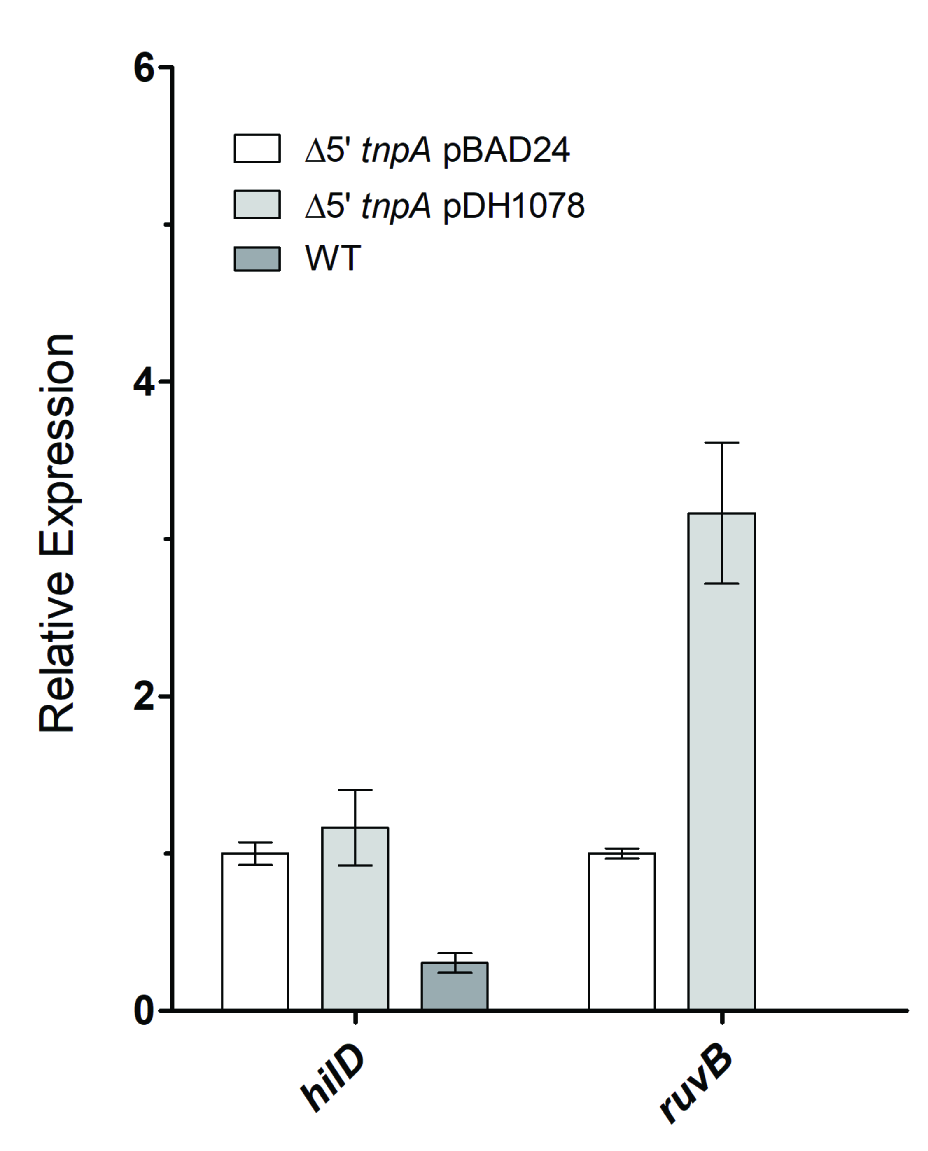


**Figure S2.** Expression of WT RuvB in the Δ*5’tnpA* strain. A plasmid (pDH1078) containing the WT *ruvB* gene fused to the pAra promoter or an empty vector control (pBAD24) was transformed into the Δ*5’tnpA* strain. Transformants were grown in LB plus or minus arabinose to an OD_600_ = 0.6 whereupon total RNA was isolated. qRT-PCR was performed to analyze the impact of *ruvB* expression on *hilD* expression. Note that results are shown for strains not treated with arabinose as the uninduced level of *ruvB* expression was most similar to the native level; defined here as the level in the Δ*5’tnpA* strain containing the empty vector control (pBAD24). Error bars show the standard deviation for three independent clones.

**Figure S3.** Growth curves for WT and Δ*5’tnpA* strains. Shaded rectangles define ranges of A_600_ values used for harvesting cells at the specific indicated growth phases for the two strains. The results represent the average +/- SD of 3 biological replicates of each strain. Lag, lag phase; EE, early exponential phase; ME, mid exponential phase; LE, late exponential phase; ES, early stationary phase; MS, mid stationary phase; LS, late stationary phase.


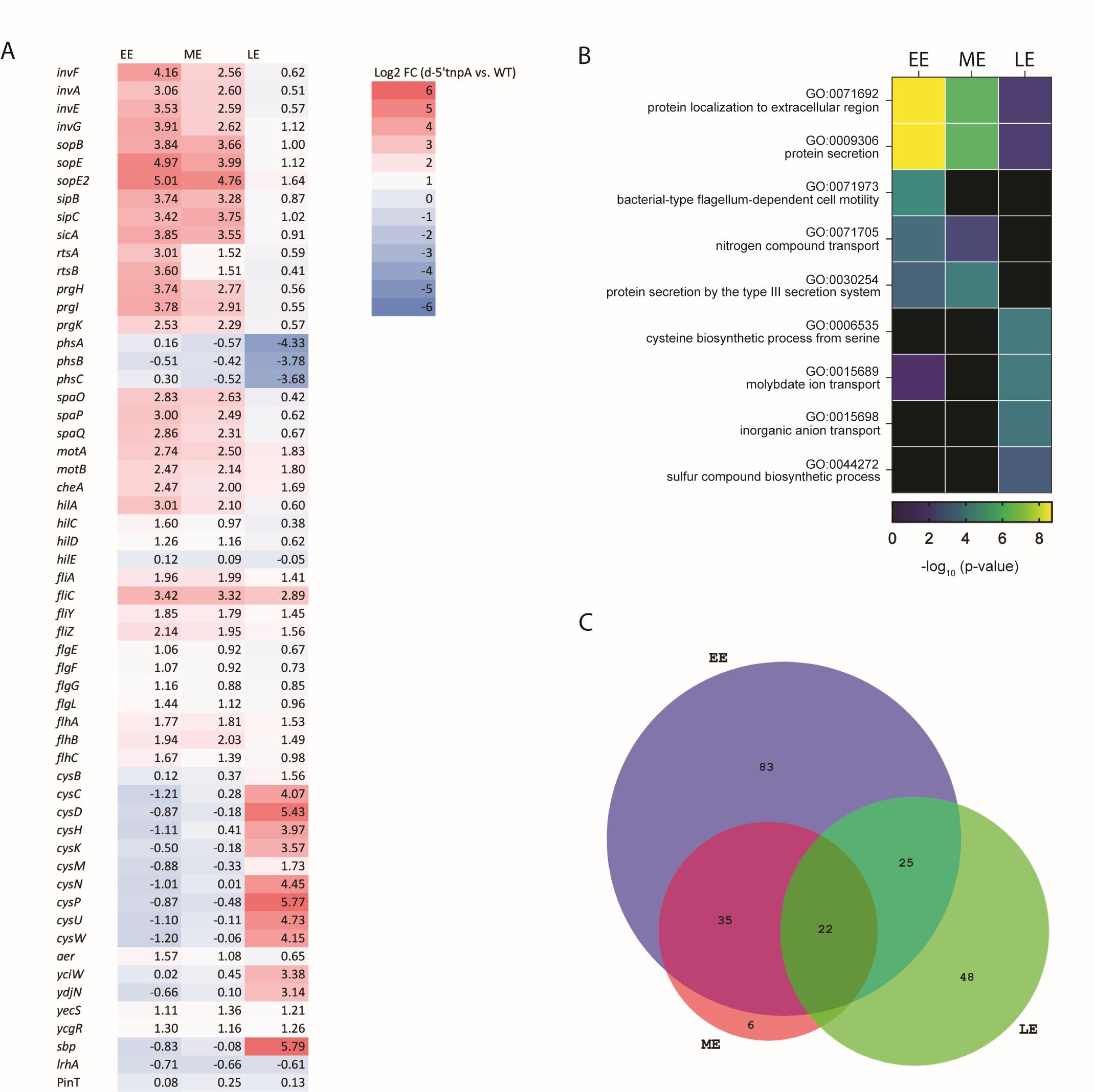


**Figure S4.** (A) Heat map visualization of the same RNA-seq data as in panels A-C of Figure 2, summarizing differential expression of genes discussed in the text. (B) Gene ontology (GO) analysis for the comparative RNA-seq data shows the most enriched GO terms in Δ*5’tnpA* compared to wild-type. (C) Venn diagram illustrating the overlap of differentially expressed genes between distinct growth phases. For the complete comparative gene expression analysis dataset, please refer to Supplementary Table S3 and Figure S5.


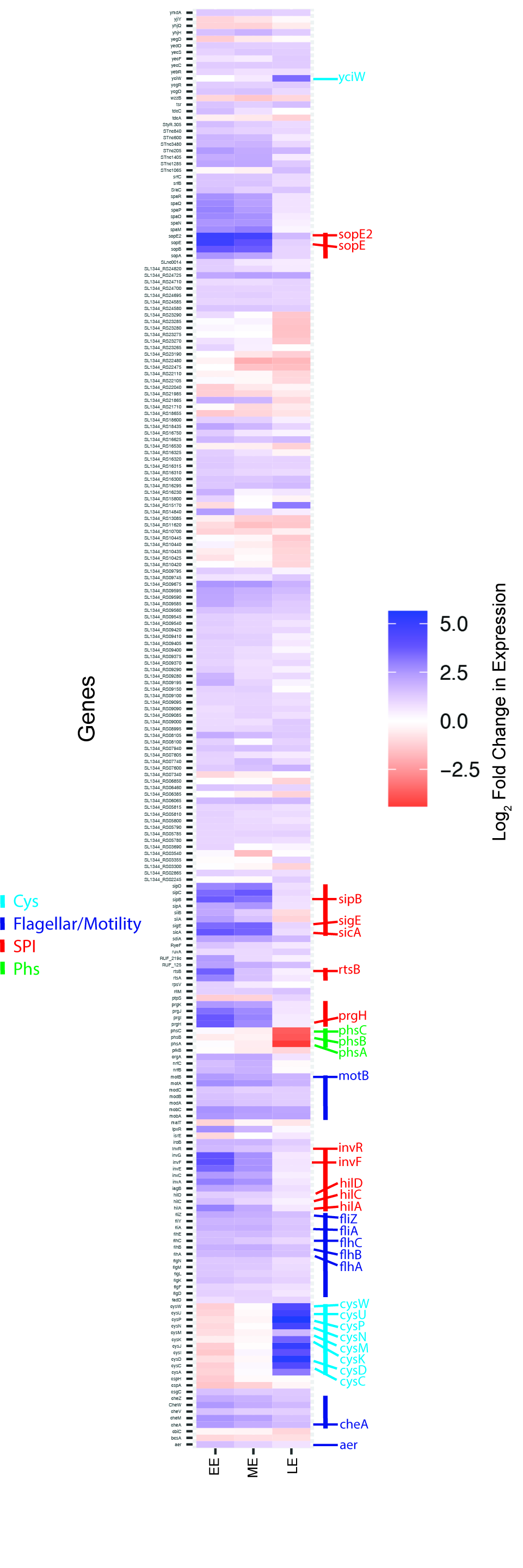


**Figure S5**. Heat map summarizing all of the genes from comparative RNA-seq showing differential levels of expression of 2-fold or more. Color coding defines genes in each of the indicated regulons.


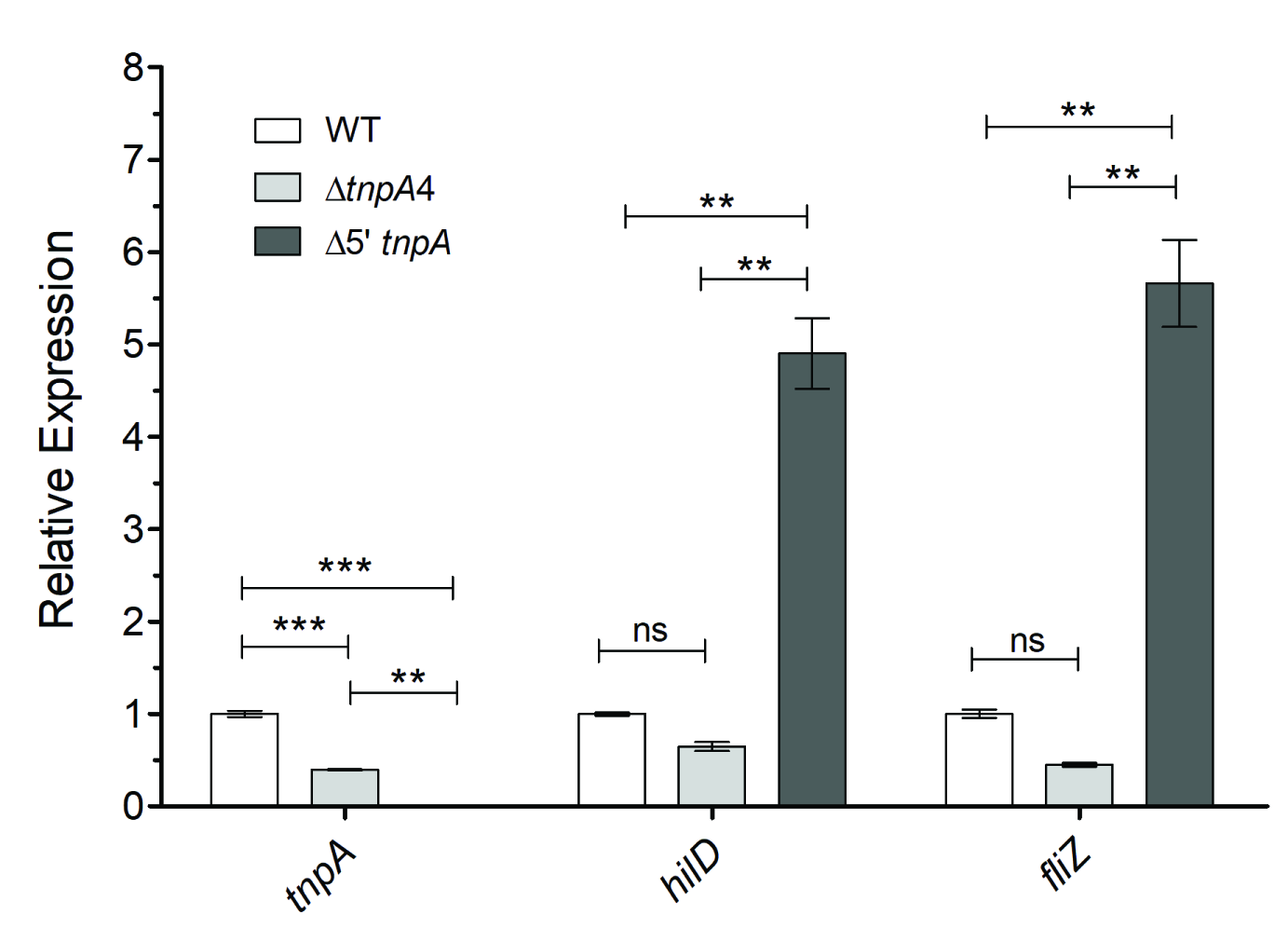


**Figure S6.** Impact of disruption of *tnpA4* on *hilD* and *fliZ* expression. The indicated strains were grown to ME phase whereupon total RNA was extracted and used for qRT-PCR analysis. The Δ*tnpA4* disruption is in an otherwise WT strain background. The Δ*5’tnpA* sample was used as a positive control for *hilD* expression. Note that there is no signal for *tnpA* expression in the Δ*5’tnpA* sample because we used tnpA qRT-PCR primers that detect the 5’ portion of the *tnpA* transcript. The *tnpA*, *hilD* and *fliZ* expression levels for the WT strain were set to 1. Error bars represent the standard deviation. P values (One-way ANOVA with Tukey’s multiple comparison test) are indicated as follows: ns, not statistically significant (P ≥ 0.05); ** and ***, P ≤ 0.01 and 0.001, respectively. Four independent clones were analyzed for each strain.


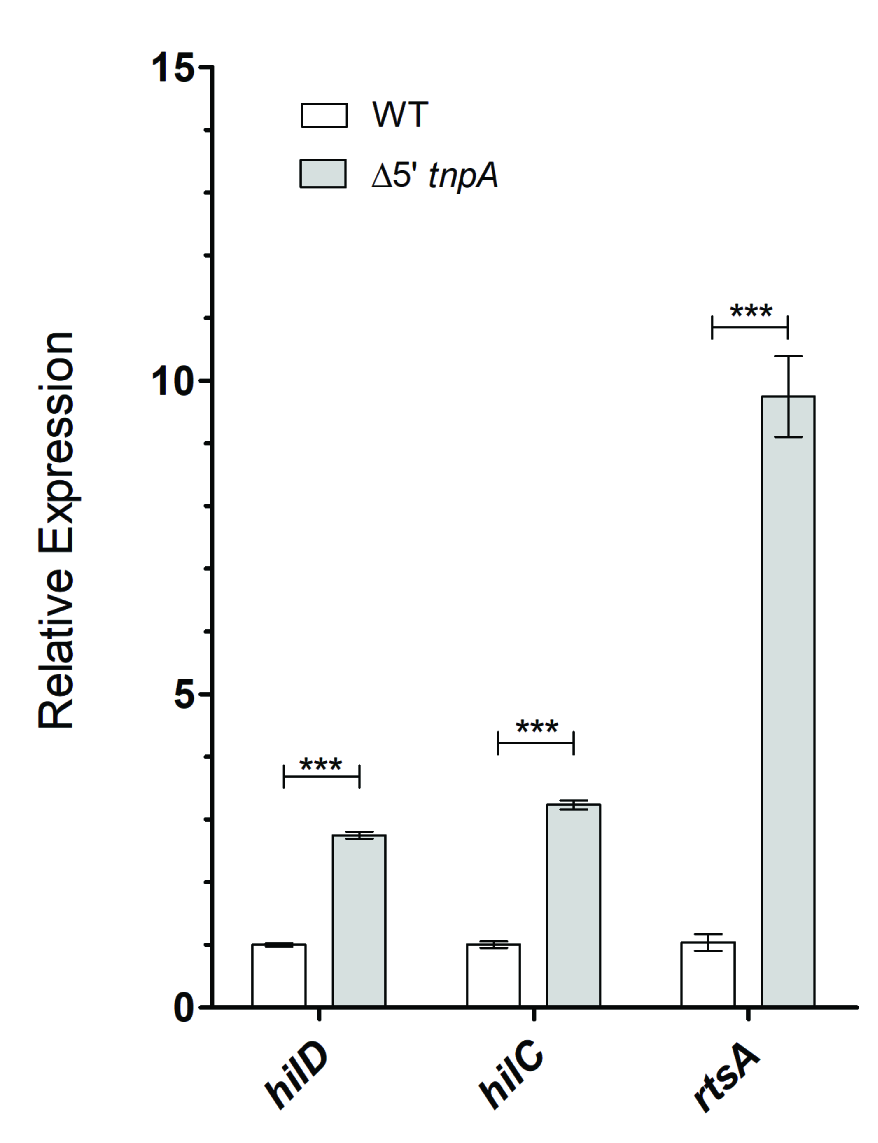


**Figure S7.** Profiling *hilD*, *hilC* and *rtsA* expression in the Δ*5’tnpA* strain. qRT-PCR was performed on RNA isolated from WT and Δ*5’tnpA* strains grown to EE phase. For each comparison the expression of the query gene in the WT strain was set at 1. Error bars show the standard deviation. P value (unpaired T-test) is indicated as follows: ***, P ≤ 0.001. Six independent clones were analyzed for each strain.


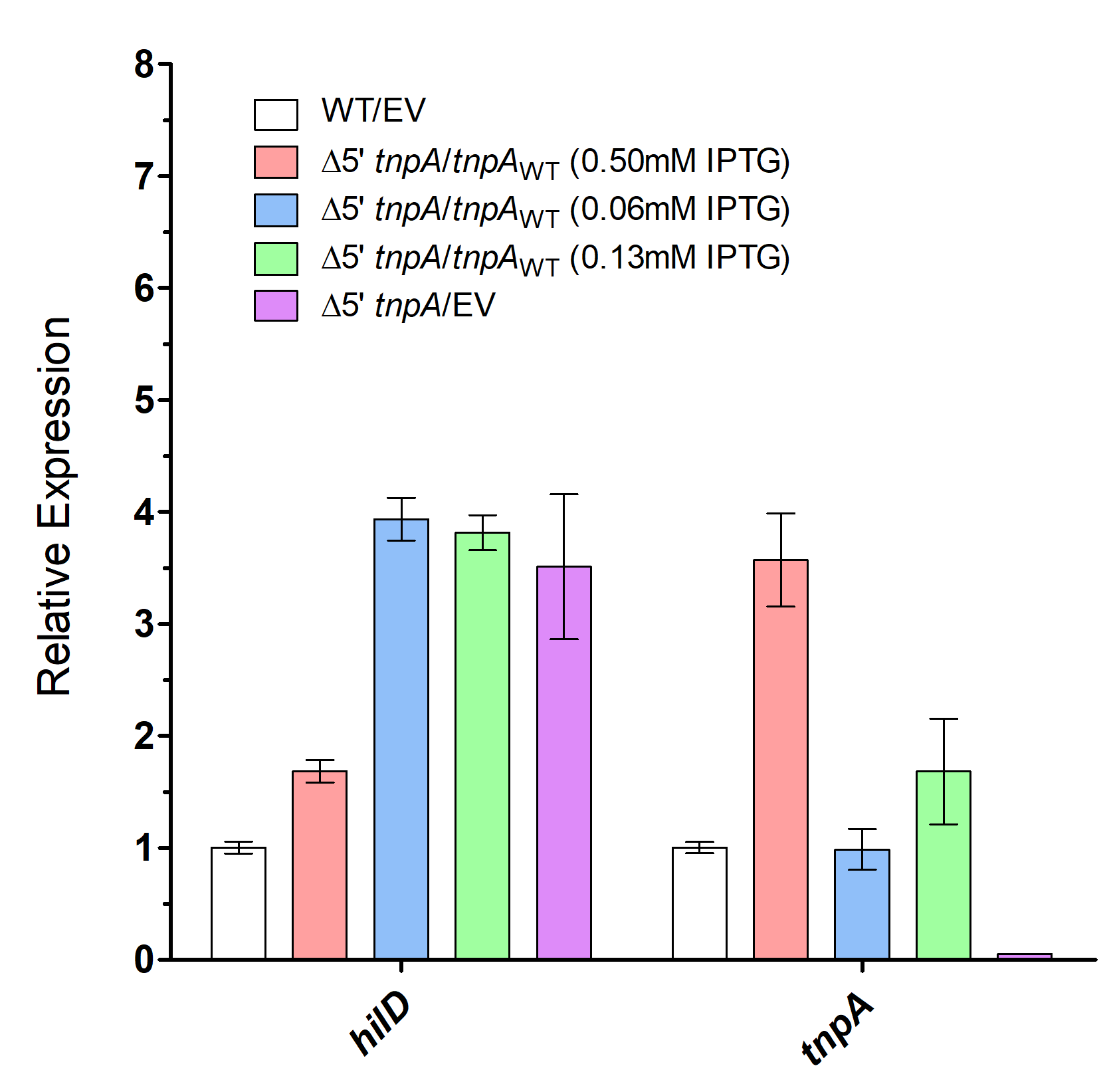


**Figure S8.** Impact of varying *tnpA* expression on *hilD* expression levels. qRT-PCR analysis of the indicated strains transformed with a *tnpA*_WT_ plasmid (pDH968) or empty vector (EV) control. After subculture, strains were grown to EE phase in the presence or absence of the indicated IPTG concentration. The expression level of the WT strain with EV control plasmid was set to 1. Error bars show standard deviation. Three independent clones were analyzed for each strain.


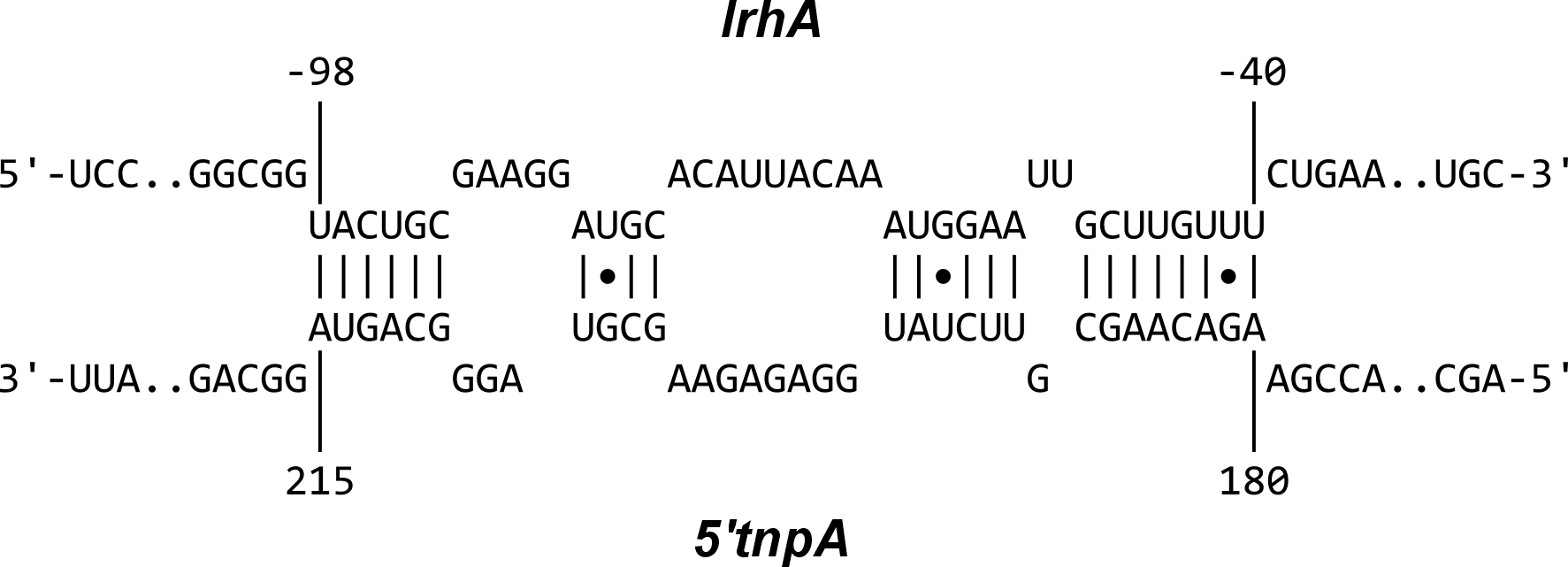


**Figure S9.** IntaRNA pairing prediction for *5’tnpA* and *lrhA* transcripts. Vertical lines and dots indicate canonical or wobble base-pairs, respectively, predicted for the *lrhA* 5’UTR (numbers relative to the translational start codon) and a segment of *tnpA* encompassing nt 180-215.


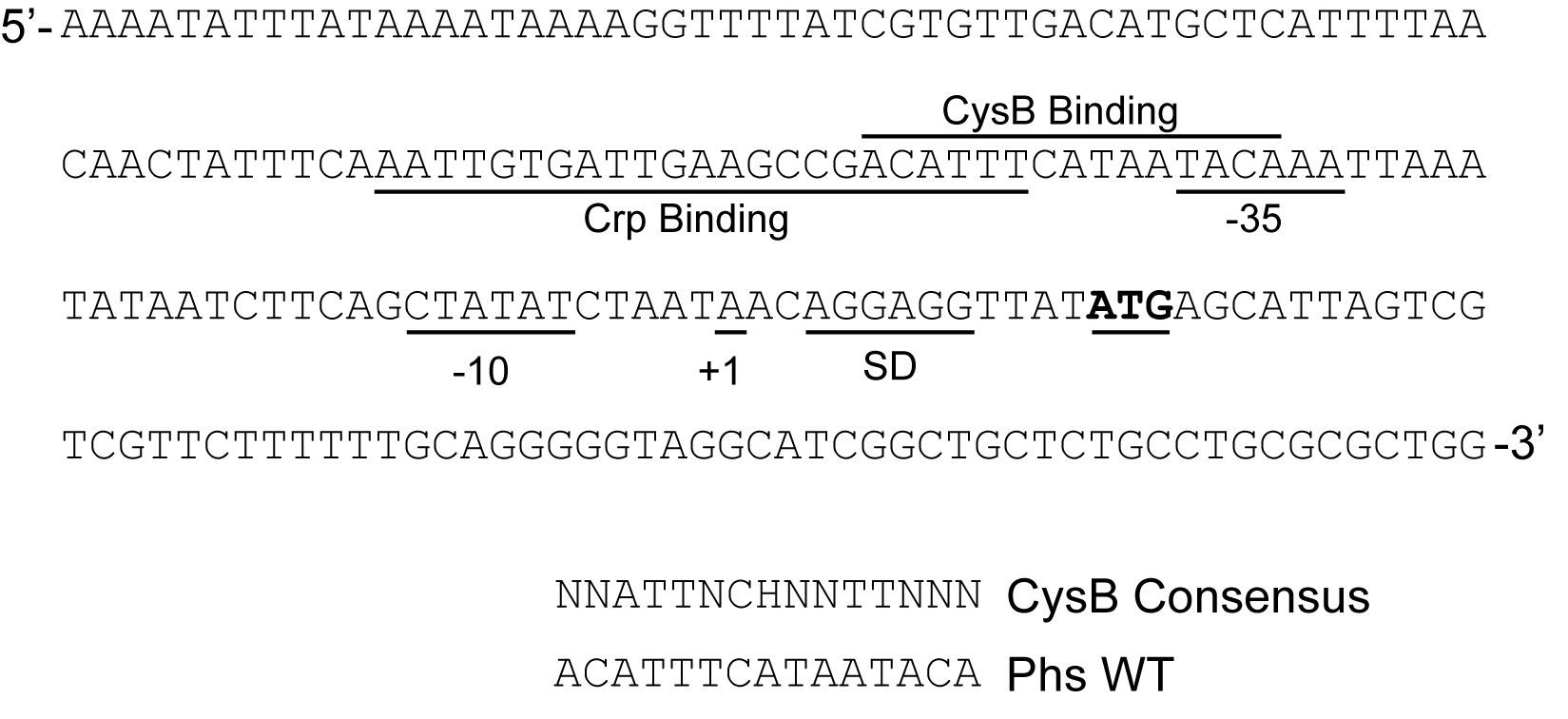


**Figure S10.** Sequence map of the *phsABC* promoter. DNA sequence of the *phsABC* operon promoter region is shown. Positions of the Crp-cAMP and putative CysB binding sites are indicated as well as the -35, -10, +1, SD (Shine-Delgarno) and start codon. DNA sequence comparisons of the putative CysB binding site and the respective consensus sequence are also shown.


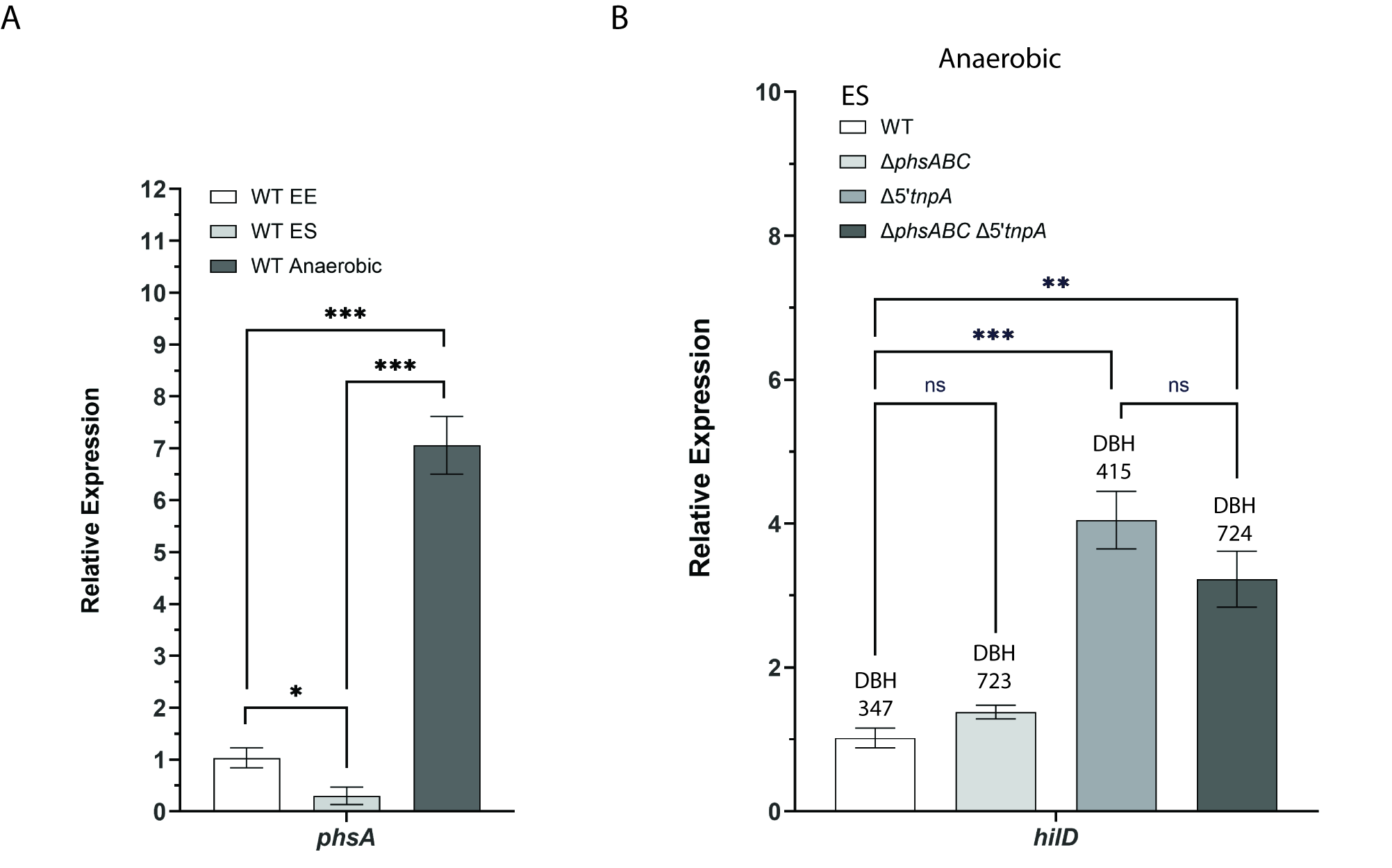


**Figure S11**. Expression profiling of *phsA* and *hilD* under aerobic and anaerobic growth. (A) The WT strain was grown to EE or ES phase under aerobic conditions or ES phase under anaerobic conditions. Total RNA was extracted and subject to qRT-PCR analysis. The *phsA* expression level for aerobic growth (EE phase) was set to 1. (B) The indicated strains were grown to ES phase under anaerobic conditions whereupon total RNA was extracted and used for qRT-PCR analysis. The *hilD* expression level for the WT strain was set to 1. Error bars show the standard deviation. P values (unpaired T-test in A and One-way ANOVA with Tukey’s multiple comparison test in B) are indicated as follows: ns, not statistically significant (P ≥ 0.05), *, ** and ***, P ≤ 0.05, 01 and 0.001, respectively. Three independent clones were analyzed for each growth condition.


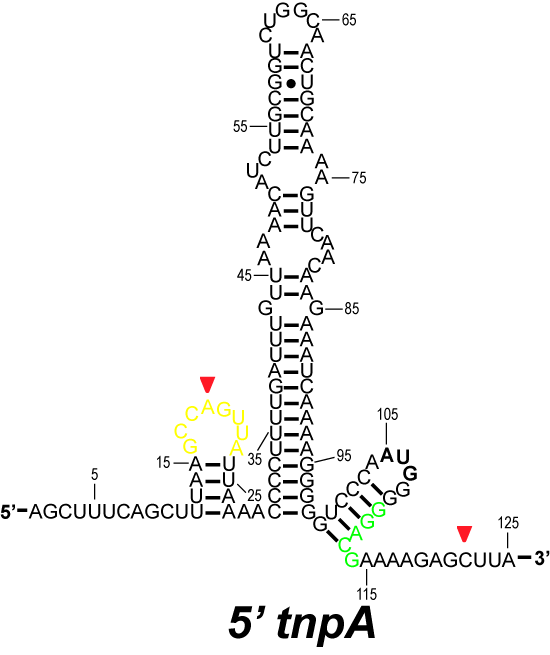


**Figure S12.** Secondary structure of 5’tnpA. The secondary structure of *5’tnpA* as determined by lead and RNase footprinting and *in silico* secondary structure predictions (Ellis *et al.*, 2015) is shown. Positions of nucleotides involved in *invF* and PinT pairing are highlighted in yellow and green, respectively. The start codon (AUG) of the *tnpA* gene is also shown as are riboendonuclease cleavage sites (red triangles).

**Supplementary References**

Datsenko, K.A., and Wanner, B.L. (2000) One-step inactivation of chromosomal genes in Escherichia coli K-12 using PCR products. *Proc Natl Acad Sci U S A* **97**: 6640–6645.

Ellis, M.J., Trussler, R.S., Charles, O., and Haniford, D.B. (2017) A transposon-derived small RNA regulates gene expression in Salmonella Typhimurium. *Nucleic Acids Res* **45**: 5470–5486.

Ellis, M.J., Trussler, R.S., and Haniford, D.B. (2015) A cis-encoded sRNA, Hfq and mRNA secondary structure act independently to suppress IS200 transposition. *Nucleic Acids Res* **43**: 6511–6527.

Fernandes, D., A., Reid, J., Macklaim, M., J., *et al.* (2014) Unifying the analysis of high-throughput sequencing datasets: characterizing RNA-seq, 16S rRNA gene sequencing and selective growth experiments by compositional data analysis. *Microbiome* **2**: 1–13.

Fu Wang, R., and Kushner, S.R. (1991) Construction of versatile low-copy-number vectors for cloning, sequencing and gene expression in Escherichia coli (Recombinant DNA; polymerase chain reaction; deletion analysis; ampicillin resistance; single-stranded DNA; kanamycin resistance). *Gene* **100**: 195–199.

Maloy, S.R.., Stewart, V.Joseph., and Taylor, R.K.. (1996) *Genetic analysis of pathogenic bacteria : a laboratory manual*. Cold Spring Harbor Laboratory Press, .

McClure, R., Balasubramanian, D., Sun, Y., Bobrovskyy, M., Sumby, P., Genco, C.A., *et al.* (2013) Computational analysis of bacterial RNA-Seq data. *Nucleic Acids Res* **41**: 1–16.

Shivak, D.J., MacKenzie, K.D., Watson, N.L., Pasternak, J.A., Jones, B.D., Wang, Y., *et al.* (2016) A modular, Tn7-based system for making bioluminescent or fluorescent salmonella and Escherichia coli strains. *Appl Environ Microbiol* **82**: 4931–4943.
